# Mobile Telemedicine for Treating Chronic Hepatitis C Among Rural People Who Inject Drugs: A Randomized Clinical Trial

**DOI:** 10.1001/jamanetworkopen.2025.55125

**Published:** 2026-01-26

**Authors:** Peter D. Friedmann, Donna Wilson, David de Gijsel, Kerry Nolte, Jean Dejace, Randall Hoskinson, Lizbeth Del Toro-Mejias, Elyse Bianchet, Patrick Dowd, William E. Soares, Thomas J. Stopka

**Affiliations:** 1 Department of Population & Quantitative Health Sciences, University of Massachusetts Chan Medical School, Worcester; 2 Department of Healthcare Delivery & Population Sciences, University of Massachusetts Chan Medical School–Baystate and Baystate Health, Springfield; 3 Office of Research, University of Massachusetts Chan Medical School–Baystate and Baystate Health, Springfield; 4 Section of Infectious Disease and International Health, Dartmouth Hitchcock Medical Center, Lebanon, New Hampshire; 5 Better Life Partners, Watertown, Massachusetts; 6 Department of Nursing, College of Health and Human Services, University of New Hampshire, Durham; 7 Division of Infectious Disease, University of Vermont Medical Center, Burlington; 8 Department of Public Health and Community Medicine, Tufts University School of Medicine, Boston, Massachusetts

## Abstract

**Importance:**

Persons who inject drugs in the rural US have high rates of chronic hepatitis C virus (HCV) infection and poor access to HCV testing, direct-acting antiviral (DAA) treatment, and syringe services. Effective approaches to test and treat this population are needed to achieve national HCV elimination goals.

**Objective:**

To test whether a mobile telemedicine-based HCV treatment intervention increases HCV treatment initiation and viral clearance and decreases sharing of injection equipment among rural persons who inject drugs.

**Design, Setting, and Participants:**

This open-label, randomized, parallel-group clinical trial compared mobile telemedicine care (MTC) for HCV treatment with enhanced usual care (EUC), both integrated with van-based syringe services, from April 21, 2022, to September 13, 2024. Participants were persons aged 18 years or older with a history of drug injection and chronic HCV infection from 3 rural counties in New Hampshire and Vermont.

**Intervention:**

MTC consisted of DAA treatment for HCV via telemedicine along with on-demand syringe services, all on a mobile van. EUC consisted of treatment referral by van staff, with care navigation to a local or regional clinician.

**Main Outcomes and Measures:**

Primary outcomes were the proportion of participants who (1) initiated DAA treatment for HCV, (2) achieved viral clearance at the 12-week follow-up, and (3) reported no injection equipment sharing at any point after the expected treatment completion visit.

**Results:**

Of 503 prescreened individuals, 169 were eligible and 150 were randomized to MTC (n = 75) or EUC (n = 75). Participants had a mean (SD) age of 38.1 (8.1) years, 103 (68.7%) were male, 105 (70.0%) experienced homelessness, and 97 (64.7%) reported drug injection in the past 30 days. MTC participants were more likely than EUC participants to initiate DAA treatment (43 [57.3%] vs 20 [26.7%]; relative risk [RR], 2.15 [95% CI, 1.41-3.28]) and to achieve viral clearance (28 [37.3%] vs 14 [18.7%]; RR, 2.00 [95% CI, 1.15-3.49]). No effect was detected on abstention from sharing of syringes or other injection equipment at follow-up (RR, 0.95; 95% CI, 0.68-1.32).

**Conclusions and Relevance:**

In this randomized clinical trial, telemedicine for chronic HCV treatment integrated with syringe services on a mobile van was associated with improved access to HCV treatment initiation and cure for people with a history of drug injection in rural communities where HCV treatment services are scarce, suggesting optimal strategies in rural areas should include convenient, low-threshold telemedicine treatment.

**Trial Registration:**

ClinicalTrials.gov Identifier: NCT05466331

## Key Points

**Question:**

Is a mobile telemedicine-based intervention for hepatitis C virus (HCV) infection associated with increased HCV treatment initiation and viral clearance and reduced injection equipment sharing among rural persons who inject drugs?

**Findings:**

In this randomized clinical trial of 150 rural persons with chronic HCV infection and a history of drug injection, a mobile telemedicine-based HCV intervention, compared with enhanced usual care, was associated with significantly greater initiation of HCV treatment (57% vs 27%) and viral clearance (37% vs 19%) but not reduced equipment sharing.

**Meaning:**

Mobile telemedicine may be a reasonable approach to increase uptake of HCV treatment among rural persons who inject drugs.

## Teaser

This randomized clinical trial investigates whether a mobile telemedicine-based intervention for chronic hepatitis C virus infection among rural people who inject drugs increases treatment initiation and viral clearance and reduces sharing of injection equipment.

## Introduction

Over the past 2 decades, hepatitis C virus (HCV) prevalence has increased among people who inject drugs in US rural areas.^1,2,3,4,5^ These populations have experienced HCV and HIV outbreaks^1,6,7,8,9^ and HCV prevalence as high as 60%.^5^ Harm reduction strategies, including provision of new injection equipment via syringe services programs (SSPs), may reduce HCV transmission,^10,11^ but the evidence is mixed^12^ and the relative dearth of brick-and-mortar SSPs in rural areas limits their impact.^13,14^

Treatment of HCV among people who inject drugs is a key strategy to achieving HCV elimination, yet access to HCV treatment is also limited in rural regions.^15,16^ Numerous studies have demonstrated that timely, on-site, low-barrier care is superior to referral as a mechanism to ensure people who inject drugs obtain needed HCV care in the context of harm reduction and opioid treatment programs, mostly in urban settings.^17,18^ The addition of telemedicine to mobile harm-reduction services or substance use treatment can extend the delivery of HCV treatment to rural people who inject drugs.^19,20^

The first phase of this project, part of the National Institutes of Health (NIH)–funded Rural Opioid Initiative, uncovered high levels of injection-related risk,^21^ elevated HCV infection rates, and limited access to sterile injection equipment and HCV treatment among people who inject drugs in rural New England.^22,23,24^ This article reports the findings from the second phase: a clinical trial to compare HCV treatment via telemedicine on a mobile van with enhanced usual care (EUC) referral as strategies to deliver HCV treatment to people who inject drugs in these rural areas.

## Methods

### Study Design

**Figure 1.**
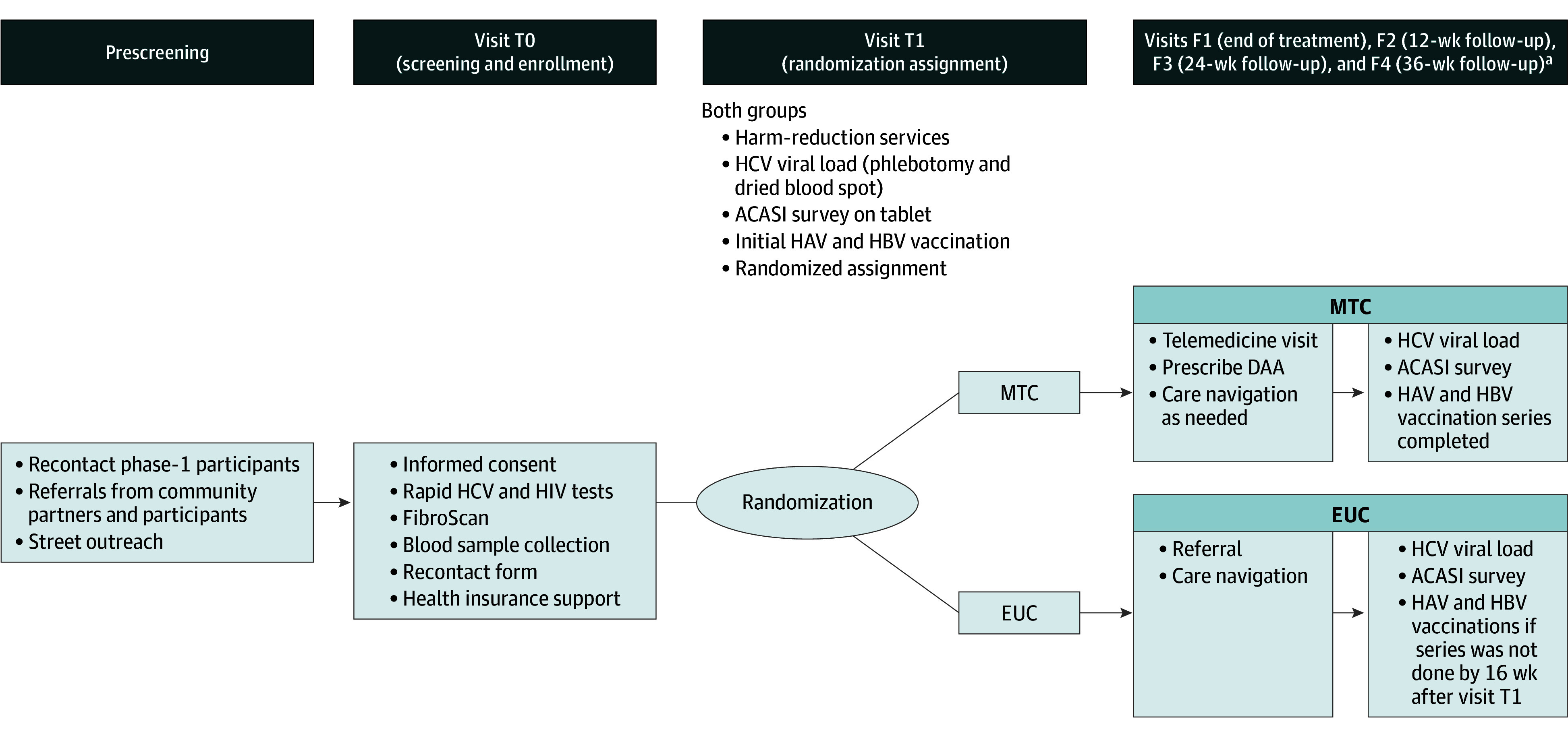
Diagram of the Study Design ACASI indicates audio computer-assisted self-interview; DAA, direct-acting antiviral; EUC, enhanced usual care; HAV, hepatitis A virus; HBV, hepatitis B virus; HCV, hepatitis C virus; MTC, mobile telemedicine care. ^a^Follow-up visits occurred 16, 28, 40, and 52 weeks after visit T1 for participants who did not initiate treatment.

This open-label randomized parallel-group superiority clinical trial examined the effectiveness of a mobile HCV treatment intervention for rural people who inject drugs (Figure 1 and protocol in Supplement 1).^25^ Participants were randomized to either (1) mobile telemedicine care (MTC) for HCV, in which a mobile van with telemedicine brought HCV treatment directly to people who inject drugs in rural communities, or (2) EUC, in which a mobile team provided care navigation and facilitated referral to a local or regional HCV treatment clinician. We hypothesized that compared with EUC, MTC would be associated with higher rates of direct-acting antiviral (DAA) treatment initiation, HCV viral clearance at the 12-week follow-up (ie, sustained virologic response 12 weeks posttreatment for those who had completed treatment), and reduced injection risk behavior, as successfully treated individuals would seek to avoid reinfection.^26,27^ Community engagement studios elicited phase 1 participants’ and stakeholders’ input into the study design and procedures.^28^ The Baystate Medical Center institutional review board approved the protocol (Supplement 1), and written informed consent was obtained from all participants. This study followed the Consolidated Standards of Reporting Trials (CONSORT) 2025 guideline.^29^

### Setting and Participants

Recruitment occurred on a mobile van parked near areas where possible participants congregated in Keene (Cheshire County), New Hampshire, and Brattleboro (Windham County) and Bennington (Bennington County), Vermont, from April 21, 2022, through January 11, 2024; the last follow-up was completed September 13, 2024. Research staff determined initial eligibility based on self-report: current or past history of drug injection, health insurance that would cover DAA medication, residence in one of the study counties and plan to remain in the study region for 12 months or longer, age of 18 years or older, ability to speak English, ability to voluntarily provide informed consent, acceptance of randomization, no previous treatment for HCV, and no current pregnancy or intent to conceive. Locator criteria that required naming 3 contacts were amended on August 1, 2022, to allow enrollment of socially isolated persons. Exclusion criteria were inability to obtain an initial venous blood sample; undetectable HCV RNA; positive hepatitis B surface antigen (HBsAg) status; significant kidney failure (estimated glomerular filtration rate of 30 mL/min/1.73 m^2^ or less or receiving dialysis) or liver cirrhosis per elastography (FibroScan [Echosens]) and/or serum fibrosis panel (FibroSure [Labcorp]) blood sample test; and evidence of decompensation: observed jaundice, self-reported increasing abdominal size and leg edema, confusion, or self-reported history of gastrointestinal bleeding.

### Prescreening and Initial Eligibility

Individuals who met initial eligibility criteria were invited to the van (visit T0) for eligibility screening and enrollment (Figure 1). Enrollment entailed receipt of a detailed explanation of the study, provision of informed consent, and point-of-care tests for HCV and HIV antibodies. Persons with HCV antibody present had phlebotomy performed for HCV RNA and genotype, guideline-based pretreatment laboratory tests (complete blood cell count, comprehensive metabolic panel, hepatitis A virus [HAV] and hepatitis B virus [HBV] serology, and β–human chorionic gonadotropin), and van-based elastography (or a serum fibrosis panel if elastography could not be completed). If a consenting participant was uninsured, study staff assisted with health insurance enrollment prior to randomization.

Study staff invited enrolled individuals to visit T1 (1-2 weeks after visit T0). Ineligible persons received paper laboratory results, a referral for services, and an invitation to receive harm-reduction supplies from the van for the duration of the study. All enrolled participants also received education about injection risk behavior and overdose prevention as part of routine harm-reduction services. A study physician (P.D.F., D.d.G., or J.D.) or nurse practitioner assessed participants with findings suggestive of fibrosis for decompensation; those with decompensated cirrhosis were excluded and referred to a hepatologist. Eligible participants received a baseline audio computer-assisted self-interview (ACASI), which collected demographics, including race and ethnicity (assessed as part of standard NIH research practice; race categories were Asian or Pacific Islander, Black, White, multiracial, and other [responses that did not fit into the other categories], and ethnicity categories were Hispanic and non-Hispanic), and relevant medical history via self-report. After initial vaccination for HAV and HBV if they lacked serologic immunity, participants were randomized.

### Randomization

At visit T1, eligible participants were randomized 1:1 to MTC or EUC. Computerized block randomization stratified by study site (Cheshire, New Hampshire; Windham, Vermont; and Bennington, Vermont), gender (male, female, or transgender), age (≤35 or >36 years), and injection drug use in the past 30 days (yes or no). The block sizes ranged from 4 to 20; size was based on the relative size of the participant subgroup in the phase I study. The study statistician (D.W.) generated the random allocation sequence in Stata, version 16 (StataCorp LLC), and uploaded it to a tool that she developed in REDCap. At visit T1, research staff (E.B. or P.D.) met with each participant, entered the 4 strata values, and clicked “randomize,” and the REDCap program provided the group assignment. Over the course of recruitment, participants and study staff expressed concern about intimate injection partners being assigned to different groups in terms of fairness and risk of reinfection; hence, an amendment on June 12, 2023, jointly assigned eligible partners of randomized participants with a current risk of transmission to the same study condition as their significant other.

### Study Conditions

A medical assistant with phlebotomy skills and 2 research coordinators (E.B., P.D.) with experience working with people who inject drugs staffed the van. Van personnel collaborated with local harm-reduction agencies to distribute syringes and harm-reduction supplies to participants and facilitate HCV testing and telemedicine treatment. Participants received monetary compensation for their time for screening, study visits, and subsequent laboratory testing (eTable 1 in Supplement 2).

#### EUC Control

At visit T1, participants assigned to EUC received a copy of their test results, a record of the initial dose of HBV and HAV vaccination, and care navigation to help arrange referral for HCV treatment. Van staff (ie, research coordinators and a medical assistant) delivered care navigation via a team approach that focused on linkage to HCV care and establishment of a release of information so the van team could communicate with clinicians. Care navigation also included an assessment of social determinants of health and referral of participants to available resources (eg, medications for opioid use disorder [MOUD], primary care, housing, transportation, and employment). EUC participants had 1 van visit for care navigation 4 weeks after visit T1 to check on HCV treatment status and other needs. EUC participants could access care navigation for the duration of HCV treatment or, for those who did not initiate treatment, for up to 16 weeks. All participants were offered initial vaccinations for HAV and HBV. Provision of the rest of the vaccine series was part of the MTC intervention, and completion of the vaccination series was a secondary outcome. For the EUC group, study personnel were ethically concerned at follow-up visits that EUC participants had not yet received the second vaccination in the series; hence, a study amendment on June 12, 2023, considered any EUC participant who had not completed the vaccination series by 16 weeks after visit T1 as having vaccination completion failure for the purposes of the secondary outcome measurement, which allowed the van team to offer to complete the series.

#### MTC Intervention

Participants assigned to MTC received a telemedicine consultation with a physician or nurse practitioner and were offered DAA treatment at visit T1, with van staff present to provide technical and social support as needed. The pangenotypic regimen containing glecaprevir-pibrentasvir was preferred, with treatment duration of 8 weeks in the absence of cirrhosis or 12 weeks in the presence of cirrhosis. Patients with mild-to-moderate kidney disease were offered pangenotypic sofosbuvir-velpatasvir for 12 weeks. Study staff assisted the patients in working with a specialty pharmacy and provided as-needed care navigation and coordination through the end of treatment. Check-in visits with the study clinician were scheduled as needed after treatment initiation to monitor response and safety and order additional medication. Follow-up HBV and HAV vaccines were administered per protocol.

### Follow-Up

Participants were asked to complete 4 follow-up visits: at end of treatment (week 8 or 12 after treatment initiation) and 12, 24, and 36 weeks after end of treatment. Follow-up assessments included an ACASI survey and HCV RNA testing via phlebotomy and capillary dried blood spot (DBS) at the first follow-up visit. Because of the demands of the COVID-19 pandemic, our original state laboratory partner had to withdraw from the project. The study contracted with a commercial laboratory (Molecular Testing Labs^30^) that had already performed its own internal validation; thus, a study amendment on December 15, 2023, allowed DBS alone for HCV RNA testing at follow-up when venous sampling was unobtainable. To allow 2 months’ time for the EUC group to obtain an initial appointment after referral and to align the follow-up times between the study groups, individuals who did not initiate HCV treatment had follow-up visits timed as if they had started 8 weeks of treatment 8 weeks after T1. In other words, for those who did not initiate treatment, follow-up occurred approximately 16, 28, 40, and 52 weeks after the T1 visit.

### Measures

The ACASI at baseline and at each follow-up visit assessed demographics, substance use, injection behavior (including syringe sharing), overdose history, substance use treatment, self-reported infectious disease and health status, and access to harm-reduction services (eg, SSPs, naloxone), MOUD, and health care services. The ACASI used harmonized measures developed as part of the Rural Opioid Initiative; for injection risk behavior, items were derived from the National Institute on Drug Abuse’s Seek, Test, Treat and Retain data collection and harmonization initiative.^31^

### Outcomes

Primary outcomes were the proportion of people who inject drugs who (1) initiated DAA treatment for HCV, assessed through the ACASI question “Have you started HCV treatment since your last visit? (yes or no),” asked at each follow-up visit; (2) achieved viral clearance at the follow-up 12 weeks after end of treatment for those treated (ie, sustained virologic response 12 weeks posttreatment) or at week 28 after visit T1 for those who never initiated treatment—this outcome could be assessed at subsequent follow-up visits if this visit was missed; or (3) reported no syringe or injection equipment sharing in the prior 30 days at any follow-up visit. The harmonized instrument asked separate questions about sharing of syringes and other injection equipment (cottons, cookers, spoons, etc). Although not specified in the original analysis plan, given the high risk of HCV transmission when sharing any injection equipment, we combined these items as a single dichotomous equipment-sharing variable at the time of analysis.

### Statistical Analysis

Descriptive analyses generated standard measures of central tendencies for continuous variables and frequencies and percentages for categorical measures. Baseline statistics were calculated for randomized participants and for participants with follow-up data. We conducted a comparison of baseline measures to assess potential differences between participants who were and were not lost to follow-up.

The original sample size (N = 220) calculated in early 2020 provided 80% or greater power (2-sided *P* < .05) to detect a medium effect size for each primary outcome (protocol in Supplement 1). Unexpected pandemic-related travel restrictions and cross-border tax issues delayed the initiation of recruitment by over 2 years and impeded full accrual prior to the end of the NIH award period. As a result, the planned target for interim analysis (50% of 220 participants completing the 12-week follow-up visit) (protocol in Supplement 1) was not reached, and interim analyses were not performed.

The main approach was intention to treat. Several factors caused missing data, including participant incarceration, housing or other environmental insecurities, study nonadherence, death, or item nonresponse. Analyses examined the impact of missingness in 3 ways. First, for the intention-to-treat models, we treated missing outcome data as treatment failure and modeled outcomes as either treatment success (initiated DAA treatment, had viral clearance) or not (did not initiate treatment, did not have viral clearance, were missing outcome data, or were lost to follow-up). These models thus included all randomized participants and had no missing outcomes. Second, we used nonimputed data to fit unadjusted and adjusted complete case models. Participants who had missing outcomes were dropped from the analysis. Third, we used multiple imputation for missing outcomes. We created 10 imputed datasets in which each outcome was imputed using fully conditional specification in SAS PROC MI (SAS, version 9.4 [SAS Institute Inc]). A logistic regression model used study group, study site, gender, age, past overdose, recent homelessness, injection drug use, syringe sharing, and opiate use at baseline to impute DAA initiation. The imputed DAA initiation variable was then used to impute the viral clearance variable. We ran logistic regression on the 10 imputed datasets, assessing the association of the intervention group with each outcome, and results were then pooled using SAS PROC MIANALYZE (SAS, version 9.4). We fit unadjusted models and explored the impact of adding covariates, including stratification variables used during randomization and any baseline variables with possible treatment-group or follow-up imbalances. Covariates used included site, gender, age, syringe sharing, and MOUD treatment and their interaction with treatment group. Due to small sample size, we used a cutoff of 2-sided *P* ≤ .30 for inclusion in adjusted models. Widths of 95% CIs were not adjusted for multiple comparisons and should not be used in place of hypothesis testing.

## Results

**Figure 2.**
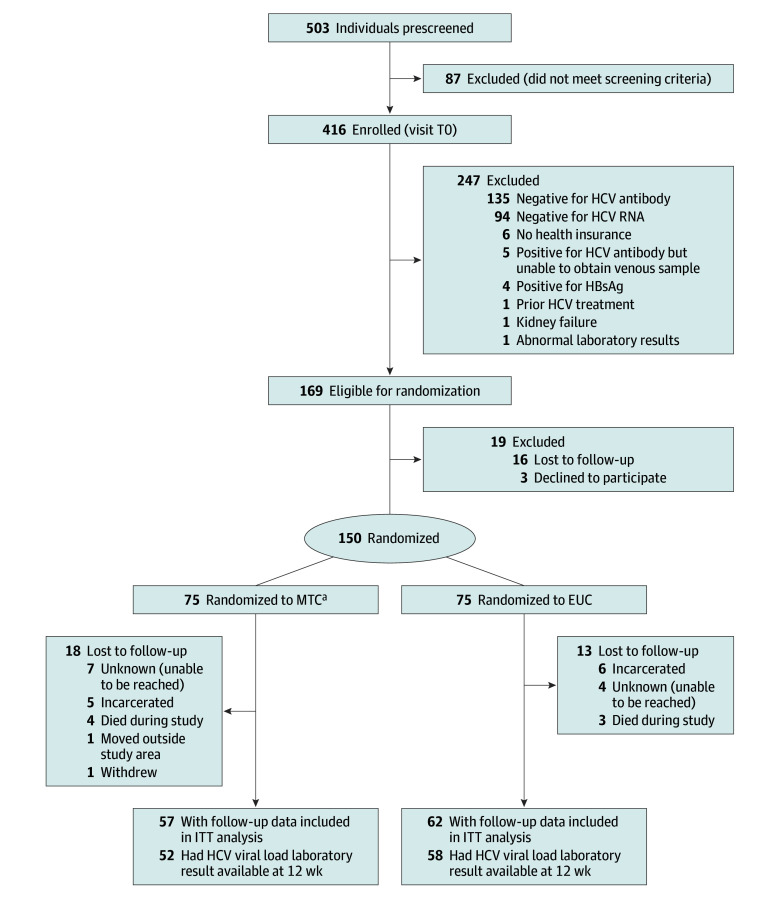
CONSORT Diagram EUC indicates enhanced usual care; HBsAg, hepatitis B surface antigen; HCV, hepatitis C virus; ITT, intention to treat; MTC, mobile telemedicine care. ^a^For 2 enrolled participants (both in the MTC group), an eligible partner for whom there was clear risk of transmission was assigned to receive treatment in the same study condition as their partner.

Of 503 prescreened individuals, 169 were eligible and 150 were randomized, 75 each to MTC and EUC (Figure 2). The randomized participants were recruited from Cheshire County, New Hampshire (51 [34.0%]), as well as Windham County (83 [55.3%]) and Bennington County (16 [10.7%]) in Vermont. The overall cohort’s mean (SD) age was 38.1 (8.1) years; 44 (29.3%) were female, 103 (68.7%) were male, 1 (0.7%) was transgender, and 2 (1.3%) had missing gender data. One participant (0.7%) was Asian or Pacific Islander, 2 (1.3%) were Black, 134 (89.3%) were White, 8 (5.3%) were multiracial, 3 (2.0%) were other race, and 2 (1.3%) had missing race data. Eight (5.3%) were Hispanic, 138 (92.0%) were non-Hispanic, 2 (1.3%) had unknown ethnicity, and 2 (1.3%) had missing ethnicity data. Almost three-quarters of participants (105 [70.0%]) had been unhoused in the previous 6 months. Approximately two-thirds reported having overdosed in the past (102 [68.0%]) and having recently injected drugs (97 [64.7%]), and 105 (70.0%) reported using opioids within 30 days of study enrollment. The study groups were balanced across measures except for syringe sharing; EUC group participants were more likely to report sharing at baseline compared with MTC participants (Table 1). The distribution of baseline measures for participants with follow-up mirrored that of all randomized participants.

**Table 1.** Baseline Characteristics by Study Arm of All Randomized Participants and Those With Follow-Up Data Only, Rural Northern New England, 2022 to 2024

Characteristic	All randomized participants (N = 150), No. (%)		Participants with follow-up data (n = 119), No. (%)	
	MTC (n = 75)	EUC (n = 75)	MTC (n = 57)	EUC (n = 62)
Site				
Cheshire County (Keene), New Hampshire	25 (33.3)	26 (34.7)	18 (31.6)	19 (30.6)
Windham County (Brattleboro), Vermont	40 (53.3)	43 (57.3)	34 (59.6)	39 (62.9)
Bennington County (Bennington), Vermont	10 (13.3)	6 (8.0)	5 (8.8)	4 (6.5)
Age, mean (SD), y	38.2 (9.1)	38.0 (7.2)	39.3 (9.1)	38.4 (7.8)
Gender				
Female	22 (29.3)	22 (29.3)	19 (33.3)	20 (32.3)
Male	50 (66.7)	53 (70.7)	37 (64.9)	42 (67.7)
Transgender	1 (1.3)	0	1 (1.8)	0
Missing	2 (2.7)	0	0	0
Race				
Asian or Pacific Islander	0	1 (1.3)	0	1 (1.6)
Black	0	2 (2.7)	0	2 (3.2)
White	66 (88.0)	68 (90.7)	51 (89.5)	56 (90.3)
Multiracial	6 (8.0)	2 (2.7)	5 (8.8)	1 (1.6)
Other^a^	1 (1.3)	2 (2.7)	1 (1.8)	2 (3.2)
Missing	2 (2.7)	0	0	0
Ethnicity				
Hispanic	4 (5.3)	4 (5.3)	3 (5.3)	3 (4.8)
Non-Hispanic	68 (90.7)	70 (93.3)	53 (93.0)	58 (93.5)
Not known	1 (1.3)	1 (1.3)	1 (1.8)	1 (1.6)
Missing	2 (2.7)	0	0	0
Educational level				
Less than high school	25 (33.3)	18 (24.0)	19 (33.3)	15 (24.2)
High school diploma or GED	31 (41.3)	39 (52.0)	25 (43.9)	33 (53.2)
Some college	11 (14.7)	15 (20.0)	9 (15.8)	11 (17.7)
Associate’s degree, trade, or technical	2 (2.7)	3 (4.0)	1 (1.8)	3 (4.8)
Bachelor’s degree or higher	3 (4.0)	0	2 (3.5)	0
Missing	3 (4.0)	0	1 (1.8)	0
Health insurance coverage				
Yes	71 (94.7)	70 (93.3)	56 (98.3)	58 (93.5)
No	0	4 (5.3)	3 (4.8)	0
Not known	2 (2.7)	1 (1.3)	1 (1.8)	1 (1.6)
Missing	0	0	0	2 (3.2)
Unhoused in past 6 mo				
Yes	49 (65.3)	56 (74.7)	37 (64.9)	46 (74.2)
No	23 (30.7)	19 (25.3)	20 (35.1)	16 (25.8)
Missing or declined to answer	3 (4.0)	0	0	0
Ever overdosed				
Yes	48 (64.0)	54 (72.0)	37 (64.9)	43 (69.4)
No	25 (33.3)	21 (28.0)	20 (35.1)	19 (30.6)
Missing	2 (2.7)	0	0	0
Injected within 30 d of baseline				
Yes	47 (62.7)	50 (66.7)	37 (64.9)	44 (71.0)
No	26 (34.7)	25 (33.3)	20 (35.1)	18 (29.0)
Missing	2 (2.7)	0	0	0
MOUD within 30 d of baseline				
Yes	24 (32.0)	31 (41.3)	16 (28.1)	26 (41.9)
No	47 (62.7)	42 (56.0)	40 (70.2)	34 (54.8)
Not known	1 (1.3)	2 (2.7)	1 (1.8)	2 (3.2)
Missing	3 (4.0)	0	0	0
Reported shared syringes or equipment within 30 d of baseline				
Yes	20 (26.7)	28 (37.3)	16 (28.1)	22 (35.5)
No	52 (69.3)	47 (62.7)	41 (71.9)	40 (64.5)
Missing	3 (4.0)	0	0	0
Any opioid use within 30 d of baseline				
Yes	50 (66.7)	55 (73.3)	40 (70.2)	47 (75.8)
No	23 (30.7)	20 (26.7)	17 (29.8)	15 (24.2)
Missing	2 (2.7)	0	0	0

Abbreviations: EUC, enhanced usual care; GED, General Educational Development; MOUD, medication for opioid use disorder; MTC, mobile telemedicine care.

^a^ Two individuals (1.3%) reported “Hebrew” and 1 (0.7%), “Italian.”

A total of 119 participants (79.3%) had follow-up data. Of the 75 participants randomized to MTC, 57 (75.8%) had follow-up data, compared with 62 of the 75 randomized to EUC (82.7%). Those randomized in Cheshire (37 of 51 [72.5%]) and Windham (73 of 83 [88.0%]) counties were more likely to have follow-up data than those from Bennington County (9 of 16 [56.3%]). Randomized participants with follow-up data were older than those without follow-up data (mean [SD] age, 38.8 [83.4] vs 35.3 [6.4] years), and females were more likely than males to have follow-up data (39 of 44 [88.6%] vs 79 of 103 [76.7%]) (eTable 2 in Supplement 2). Study engagement was similar for both groups, with 62 EUC participants (82.7%) and 56 MTC participants (74.7%) reporting whether they had initiated DAA treatment and 59 EUC participants (78.9%) and 51 MTC participants (68.0%) reporting viral clearance results. Deaths occurred in 4 MTC participants (5.3%) and 3 EUC participants (4.0%) (Figure 2).

### DAA Initiation

MTC participants had higher rates of DAA treatment initiation than EUC participants (Table 2). For the MTC group, 43 participants (57.3%) reported initiating DAA treatment compared with 20 EUC participants (26.7%), with 19 MTC participants (25.3%) and 13 EUC participants (17.3%) with missing data or loss to follow-up (Table 2). In intention-to-treat models (Table 3), MTC participants experienced increased likelihood of DAA initiation (relative risk [RR], 2.15; 95% CI, 1.41-3.28).

**Table 2.** Crude Outcome Frequencies With Denominators

Outcome	Participants, No./total No. (%)							
	Mobile telemedicine HCV care				Enhanced usual care			
	Intention to treat^a^^,^^b^	Missing response^b^^,^^c^	Complete case^d^	Multiple imputation^e^	Intention to treat^a^^,^^b^^,^^c^	Missing response^b^^,^^c^	Complete case^d^	Multiple imputation^e^
Initiated DAA treatment	43/75 (57.3)	19/75 (25.3)	43/56 (76.8)	56/75 (74.7)	20/75 (26.7)	13/75 (17.3)	20/62 (32.3)	24/75 (32.0)
Viral clearance^f^	28/75 (37.3)	24/75 (32.0)	28/51 (54.9)	40/75 (53.3)	14/75 (18.7)	16/75 (21.3)	14/59 (23.7)	18/75 (24.0)
No reported injection equipment sharing	35/75 (46.7)	25/75 (33.3)	35/50 (70.0)	52/75 (69.3)	37/75 (49.3)	18/75 (24.0)	37/57 (64.9)	49/75 (65.3)

Abbreviations: DAA, direct-acting antiviral; HCV, hepatitis C virus.

^a^ Participant had a positive outcome on follow-up (initiated DAA medication, had undetectable HCV RNA at 12-week follow-up [viral clearance], or reported no equipment sharing after expected treatment end). Participants with a negative or missing outcome were considered negative for the outcome.

^b^ Denominator for the column is all randomized participants for DAA initiation and HCV clearance outcomes.

^c^ “Missing response” indicates the number of participants lost to follow-up or missing the outcome value.

^d^ “Complete case” indicates the participant had the outcome value. The denominator is the number of participants with the outcome value.

^e^ “Multiple imputation” indicates combined results after multiple imputation of the outcome or predictor value.

^f^ Assessed as a negative HCV RNA result at the 12-week follow-up after treatment completion (ie, sustained viral response at 12 weeks among treated participants) or at week 28 after randomization among participants who did not initiate treatment.

**Table 3.** Predicted Probabilities and Relative Risks by Intention-to-Treat, Complete-Case, and Multiple-Imputation Approaches

	Participants, No.	Predicted probability (95% CI)^a^^,^^b^		RR (95% CI), unadjusted models^b^^,^^c^
		Mobile telemedicine HCV care	Enhanced usual care	
**Initiated DAA treatment**				
Intention to treat^d^	150	0.57 (0.47-0.70)	0.27 (0.18-0.39)	2.15 (1.41-3.28)
Complete case^e^	118	0.77 (0.66-0.89)	0.32 (0.22-0.46)	2.38 (1.61-3.51)
Multiple imputation (n = 10)^f^	Pooled	0.74 (0.61-0.87)	0.31 (0.19-0.43)	2.36 (1.48-3.75)
**Viral clearance^g^**				
Intention to treat^d^	150	0.37 (0.28-0.50)	0.19 (0.12-0.30)	2.00 (1.15-3.49)
Complete case^e^	110	0.55 (0.43-0.70)	0.24 (0.15-0.37)	2.31 (1.37-3.89)
Multiple imputation (n = 10)^f^	Pooled	0.53 (0.39-0.66)	0.24 (0.13-0.34)	2.21 (1.38-3.56)
**No reported injection equipment sharing**				
Intention to treat^d^	150	0.47 (0.37-0.59)	0.49 (0.39-0.62)	0.95 (0.68-1.32)
Complete case^e^	107	0.70 (0.58-0.83)	0.65 (0.54-0.79)	1.08 (0.83-1.40)
Multiple Imputation (n = 10)^f^	Pooled	0.70 (0.56-0.83)	0.65 (0.54-0.77)	1.07 (0.83-1.37)

Abbreviations: DAA, direct-acting antiviral; HCV, hepatitis C virus; RR, relative risk.

^a^ Estimated probabilities were from logistic regression models.

^b^ The 95% CI widths were not adjusted for multiple comparisons and should not be used in place of hypothesis testing.

^c^ RRs are from log-binomial models.

^d^ Participants had a positive outcome on follow-up (initiated DAA medication, had undetectable HCV RNA at 12-week follow-up, or reported no equipment sharing after expected treatment end). Participants with a negative or missing outcome were considered negative for the outcome.

^e^ Participants had the outcome value.

^f^ Pooled results after imputation from 10 imputed datasets using fully conditional specification.

^g^ Assessed as negative for HCV RNA at the 12-week follow-up after treatment completion (ie, sustained viral response at 12 weeks) or at week 28 after visit T1 (T1 was defined as 1-2 weeks after initial enrollment visit) among participants who did not initiate treatment.

### Viral Clearance

MTC participants had higher rates of viral clearance at the 12-week follow-up than EUC participants (Table 2). In the MTC group, 28 participants (37.3%) achieved viral clearance vs 14 (18.7%) in the EUC group, with 24 MTC participants (32.0%) and 16 EUC participants (21.3%) having missing data. Intention-to-treat models provided the most conservative effect estimates (Table 3): MTC participants experienced an increased likelihood of viral clearance compared with EUC participants (RR, 2.00; 95% CI, 1.15-3.49). Including only those who initiated DAA treatment (eTable 3 in Supplement 2), viral clearance rates did not differ among the study conditions (27 of 43 [62.8%] for MTC, 13 of 20 [65.0%] for EUC). Viral clearance rates appeared to be higher among EUC participants (11 of 15 [73.3%]) than among MTC participants (23 of 34 [67.6%]) who completed DAA treatment.

### Syringe or Injection Equipment Sharing

At baseline, 28 EUC participants (37.3%) and 20 MTC participants (26.7%) had reported recent sharing of syringes or other injection equipment (Table 1). Across all follow-up visits, 35 participants in the MTC group (46.7%) reported any sharing compared with 37 in the EUC group (49.3%), with 25 MTC participants (33.3%) and 18 EUC participants (24.0%) having missing data (Table 2). Differences were not significant for syringe or injection equipment sharing in intention-to-treat models (RR, 0.95; 95% CI, 0.68-1.32). In post hoc analyses, participants who completed HCV treatment (49 [32.7%]) (ie, by treatment received) did not have significantly greater reported abstention from equipment sharing during follow-up than those who did not complete HCV treatment (64 [42.7%]) (RR, 0.83; 95% CI, 0.62-1.11).

## Discussion

This randomized clinical trial found that HCV treatment via telemedicine integrated with mobile syringe services on a van was associated with a doubling of access to HCV DAA treatment initiation and viral clearance among rural people who inject drugs compared with referral to usual care enhanced with care navigation in rural communities.

Once DAA treatment was initiated, over 60% of participants in both groups cleared the virus at 12 weeks. This observation suggests that the higher rate of DAA initiation in the MTC group, the result of timely telemedicine treatment as opposed to referral in the EUC group, explains the difference in viral clearance between the study conditions. These findings add to the accumulating evidence that telemedicine treatment is associated with improved HCV treatment uptake and cure among rural persons who use drugs.^19,32,33^ A recent clinical trial found that peer-assisted telemedicine increased HCV treatment initiation and cure vs peer-assisted referral to local clinicians in rural Oregon.^19^ The current study’s results also contribute to the evidence that integration of HCV treatment into harm-reduction services enhances HCV treatment uptake and cure.^34,35^ Treatment as prevention is a major component of strategies to combat HIV^36,37^ but has yet to be widely implemented or evaluated for HCV. For most people living with HCV, current DAA treatments could cure their HCV infection and help to prevent downstream transmission. The 62.8% to 65.0% cure rate among rural people who inject drugs who initiated DAA treatments in the current study was substantially lower than that reported in opioid treatment programs and controlled trials^38,39^ and at the lower end of community-based cure rates for self-administration of DAA treatments among out-of-treatment people who inject drugs,^40,41^ including those in rural areas.^19^ Nonetheless, modeling studies suggest that curing even a fraction of people who inject drugs would decrease HCV incidence^42,43,44^ and may be cost-saving.^45^ These models accounted for reinfection rates, which are historically relatively low.^46^

Among urban people who inject drugs, prior work has suggested that HCV treatment was associated with decreased injection risk behavior.^26,27,47,48,49,50,51^ This study incorrectly posited that greater access to HCV treatment in the MTC group would lead to less risky injection behavior, as individuals would seek to avoid reinfection. Despite an increase in treatment uptake and cure, mobile telemedicine HCV treatment in the current study was not related to reduced sharing of injection equipment compared with enhanced usual care with equal access to harm-reduction supplies from a van. Our result is consistent with another recent rural HCV treatment study in which HCV treatment itself did not impact injection risk behavior.^35^ The relative paucity of syringe access in rural areas may make it challenging for HCV treatment to reduce risky injection behavior in the absence of peer contact encouraging safer injection practices.^35^

### Limitations

This study has several limitations. Both study groups received care navigation, harm-reduction services, HAV and HBV vaccination, and ongoing assessments from van staff, and many in the EUC group received referral to the same HCV clinicians who delivered the MTC care; these overlaps likely reduced effect differences between the study groups. In addition, lack of blinding may have biased ACASI-reported assessments, especially for injection equipment sharing. Furthermore, the van traveled to each study site once or twice per week; more frequent availability may have facilitated better follow-up rates and outcomes. The study also occurred during the COVID-19 pandemic, which hampered recruitment and likely limited care seeking and availability.

## Conclusions

In conclusion, this randomized clinical trial found that telemedicine-based HCV treatment delivered on a mobile van with syringe services was associated with increased uptake of DAA treatment and significant improvements in cure rates among rural people who inject drugs. These findings suggest that referral to local HCV treatment clinicians, even with care navigation, is suboptimal to facilitate HCV treatment for rural people who inject drugs.^52,53^ Optimal strategies should combine point-of-care HCV antibody and RNA testing; convenient, low-threshold telemedicine treatment integrated with enhanced harm-reduction or MOUD services; and peer support for HCV treatment.^19,54,55^

## Notes

Supplement 1.
Trial Protocol

Supplement 2. **eTable 1.** Schedule of Participant Payments **eTable 2.** Baseline Characteristics Comparing Randomized Participants With and Without Follow-Up Data, Rural Northern New England, 2022 to 2024 **eTable 3.** Viral Clearance at 12-Week Follow-Up by Study Condition, Initiation, and Completion of Direct-Acting Antiviral (DAA) Treatment, Rural Northern New England, 2022 to 2024

Supplement 3.
Data Sharing Statement
