# Supplementary material for: Mobile Telemedicine for Treating Chronic Hepatitis C Among Rural People Who Inject Drugs: A Randomized Clinical Trial
Source: JAMA Netw Open. 2026 Jan 26;9(1):e2555125. doi: 10.1001/jamanetworkopen.2025.55125 (PMC12836132; doi:10.1001/jamanetworkopen.2025.55125)
Supplement: Supplement 1. — Trial Protocol [file jamanetwopen-e2555125-s001.pdf]

I. Protocol Title:

Drug Injection Surveillance and Care Enhancement for Rural Northern New England (DISCERNNE)

UH3 – Phase 2 Protocol

Principal Investigators:
Peter D. Friedmann, MD, MPH, DFASAM, FACP
Thomas J Stopka, PhD, MHS

Protocol version:
~~5/15/2019~~
11/05/2021

**Glossary of Abbreviations**			
ACASI	Audio computer assisted survey instrument	ITT	Intent-to-treat
AE	Adverse event	LLD	Lower limit of detection
aOR	Adjusted odds ratio	MA	Massachusetts
BMC	Baystate Medical Center	MGH	Massachusetts General Hospital
BLP	Better Life Partners	MPI	Multiple principal investigator
BRL	Baystate Reference Laboratories	MTC	Mobile tele-HCV care (the study condition)
BUP-NX	Buprenorphine-naloxone	MOUD	Medication for opioid use disorder
CAB	Community Advisory Board	NIDA	National Institute on Drug Abuse
CAPI	Computer assisted personal interview	NIH	National Institutes of Health
CDC	Centers for Disease Control	NH	New Hampshire
CV	Coefficient of variation	OUD	Opioid use disorder
DAA	Direct-acting antiviral treatment for HCV	OR	Odds ratio
DBS	Dried blood spot	PCR	Polymerase chain reaction
DCC	Data Coordinating Center	PWID	People who inject drugs
DISCERNNE	Drug Injection Surveillance and Care Enhancement for Rural Northern New England	QALY	Quality-adjusted life-years
		RA	Research assistant
DSMB	Data safety and monitoring board	RNA	Ribonucleic acid
ED	Emergency department	ROI	Rural Opioid Initiative
EIA	Enzyme-linked immunosorbent assay	SAE	Serious adverse event
ESC	Executive Steering Committee	SSP	Syringe services program
EUC	Enhanced usual care (the control condition)	SUD	Substance use disorder
FDA	Food and Drug Administration	SVR12	Sustained virological response 12 weeks after treatment (a.k.a., cure)
GHOST	Global Hepatitis Outbreak and Surveillance Technology laboratory	TasP	Treatment as prevention
		5’UTR	5’ untranslated region
HBsAg/Ab	Hepatitis B surface antigen /antibody	UMass Chan Baystate	UMass Chan Medical School-Baystate
HAV/ HBV/ HCV	Hepatitis A, Hepatitis B, Hepatitis C virus		
HIV	Human immunodeficiency virus	VL	Viral load
HRQoL	Health-related quality of life	VT	Vermont
ID	Infectious disease		
IDU	Injection drug use		

II. SPECIFIC AIMS

The unprecedented U.S. opioid use disorder (OUD) epidemic has created the impetus for novel approaches to reduce overdose deaths and infectious diseases associated with injection drug use (IDU), including hepatitis C virus (HCV) and HIV.[1] The devastating outbreak of HIV and HCV in Scott County, Indiana, which was associated with syringe sharing and injection opioid use, has raised particular concerns about high-risk persons who inject drugs (PWID) in rural counties across the U.S. where geographic dispersion, poor public transportation, and limited public health infrastructure constrain the delivery of effective preventive and treatment services.[2] In New England's rural north, the opioid overdose epidemic has accelerated with the introduction of illicit fentanyl,[3] and HCV rates have increased dramatically.[4]

Our UG3 work over the past two years has examined the epidemiology and service environment relevant to the syndemic of injection opioid use and HCV in rural counties along the Interstate 91 corridor in Massachusetts (MA), New Hampshire (NH) and Vermont (VT). Findings suggest that these counties, especially those in NH and VT are at high risk for a Scott County-like outbreak — syringe sharing and HCV are highly prevalent, while access to clean syringes, phlebotomy services, HCV testing and treatment are limited. Other relevant lessons learned from the UG3 phase include: (1) any intervention must limit the burden on the local harm reduction and medical institutions that have limited space and resources for new programming and are straining to meet their current responsibilities; (2) any intervention siting must account for the reluctance of active PWIDs to seek care from a health system that has treated them poorly; and (3) advances like dried blood spot testing (DBS) are needed to address the difficulty of limited access to phlebotomy services among PWIDs in rural areas.

Over the 3-year UH3 phase we will integrate mobile HCV testing and treatment with expanded syringe access in order to improve uptake of HCV testing and treatment, and reduce syringe sharing behavior. The model aims to reach rural opioid injectors with HCV and to fill service gaps identified in our UG3 study around access to syringe services and HCV testing and treatment, while limiting the burden on local partners. Specific aims for the UH3 phase are to:

1. Perform a Type 1 hybrid effectiveness-implementation study to examine the effectiveness of a model of mobile telemedicine treatment for HCV integrated with syringe services programming, versus the current clinical practice of referral to a local or regional provider, enhanced with care navigation.
2. Validate the accuracy of dried blood spot (DBS) testing for HCV viral load as a potential surveillance strategy to address limited access to phlebotomy services in rural areas.

Working closely with harm reduction experts in rural communities with poor access to sterile syringes, we will employ a mobile syringe services van to expand HCV antibody and viral testing, bolster syringe access, and provide HCV telemedicine treatment. PWIDs who tested positive for HCV antibody during the UG3 phase will be re-contacted, with supplemental sampling from local referrals. All consenting PWIDs with HCV antibody present will undergo DBS fingerstick and venipuncture for HCV viremia, and, as appropriate, receive initial hepatitis B virus (HBV) and/or hepatitis A virus (HAV) vaccines. We will then randomize them (n= 220) to one of two strategies for HCV work-up and treatment among rural HCV-infected PWIDs:

(1) Enhanced Usual Care (EUC) – referral with care navigation to a local or regional HCV treatment provider.[5]
(2) Mobile tele-HCV Care (MTC) – HCV telemedicine Direct-Acting Antiviral treatment for HCV (DAA) on a mobile van.

Primary outcomes will be the proportion of PWID who initiate DAA treatment, achieve sustained virologic response at 12-weeks post-treatment (SVR12), and report no syringe sharing in the prior 30 days at 12-week follow-up. We hypothesize that, compared with EUC, mobile tele-HCV care will be associated with higher rates of DAA treatment initiation, higher rates of sustained virologic response 12-weeks post treatment (SVR12, a.k.a. cure), and lower rates of syringe sharing behavior. Secondary outcomes will examine HAV and HBV vaccination completion rates, medication for opioid use disorder (MOUD) initiation, ED visits, health-related quality of life (HRQOL) and substance use. Formative evaluation will assess barriers, facilitators, and implementation lessons-learned.[6]

If effective, this mobile model of HCV telehealth integrated with syringe services will provide a promising approach for local public health authorities seeking to curb opioid injection, syringe sharing and HCV rates in rural America, and reduce the risk environment for HIV outbreaks in those communities.

III. Background.

The syndemic of opioid use disorder, overdose and associated infections continues. From 1999 to 2014, the age-adjusted U.S. death rate from drug overdose nearly tripled, surpassing mortality from vehicular accidents or firearms.[3,8] People who inject drugs (PWID) are also at risk for acquiring and transmitting HIV and HCV through risky behaviors such as sharing drug equipment, unprotected sex while under the influence, having multiple sex partners, and exchanging sex for drugs or money.[9,10]

Syringe sharing and poor access to clean syringes are driving the high rate of HCV and other infectious complications associated with OUD. Unsafe injection practices among PWID have led to rising infectious complications from acquiring and transmitting HIV, HAV, HBV, and HCV[11,12] and dying (e.g., HCV deaths exceed HIV deaths).[13,14] Of 38,739 new HIV diagnoses in the U.S. in 2017, 6.2% were linked to IDU.[15] Among HIV-negative PWID in 20 states, 61% reported receptive sharing of injection equipment in the prior 12 months. Furthermore, equipment sharing was reported among 69% of HIV-positive PWID who were unaware of their HIV status and 53% of PWID who were aware of their HIV status.[16]

PWID communities in rural New England are at high risk for HIV and HCV outbreaks. The HIV and HCV outbreak in Scott County, Indiana, which was associated with opioid injection, syringe sharing,[2] and multiple injections during one sitting[17] raised concerns about high-risk PWID in rural areas where geographic dispersion, poor public transportation, and limited harm reduction infrastructure constrain the delivery of effective preventive and treatment services.[2] A recent HIV outbreak among PWID in the Merrimack Valley of MA, largely attributed to fentanyl injection,[18,19] and clinical advisories from MDPH regarding spikes in new HIV infections among PWID in additional communities, point to increasing public health challenges in New England.[20] An “emerging epidemic” has been detected among growing numbers of rural youth who started with oral prescription opioids or insufflated heroin and then transitioned to injection of these drugs,[21] leading to increasing prevalence of IDU in rural areas and higher rates of HCV in rural than in urban areas since 2006.[7,12] The CDC published its assessment of rural “counties at risk” as particularly vulnerable to a Scott County-like outbreak of HIV and hepatitis among PWIDs,[7] which included two Vermont counties, Essex and Windham, evaluated in our UG3 work in rural northern New England.[7] While HIV prevalence is low in Vermont, 21% of new cases in 2013 were detected in PWIDs.[22] Acute HCV rates increased by 150% between 2009 and 2013.[22] Acute HBV rates also increased from 0.3/10,000 in 2010 to 0.6/10,000 in 2015.[23]

Although no NH counties were included in the CDC’s counties at risk, this result is likely an artifact of limited reporting. New Hampshire was among the 19 states with statistically significant increases in the opioid overdose death rate from 2013 to 2014,[1] but HCV only became reportable in NH in January 2017. Our UG3 work has found high rates of syringe sharing and HCV infection rates as high as 70% among PWIDs. NH has limited rural OUD treatment, few SSPs and little harm reduction infrastructure; poor syringe access compounds NH’s rural PWIDs high risk for HIV and HCV. Notably, the Merrimack Valley in MA that has experienced a significant increase in HIV infections among PWID also spans the MA-NH border, and a number of our UG3 study participants in Southern NH mentioned traveling to the HIV hotspot in MA to obtain drugs and supplies. HCV rates and other comorbidities of drug injection have increased along similar trajectories, especially among young PWIDs.[4] These trends highlight the need to address widespread syringe sharing, expand HCV and HIV surveillance and treatment, and improve access to harm reduction services in these rural areas.[2]

Our UG3 work over the past two years in rural counties along the Interstate 91 corridor in MA, NH and VT suggest that several counties are at high risk for Scott County-like outbreaks — syringe sharing and HCV are highly prevalent, while access to clean syringes, phlebotomy services, HCV testing and treatment are limited, especially in NH and VT. Among the first 377 current PWIDs (i.e. injected in past 30 days) in our UG3 study, 68% tested positive for HCV and 70% reported sharing injection equipment, 74% injected multiple times in one sitting, 58% had overdosed at least once, and 19% reported never having received any form of addiction treatment; 79% reported having sex without a condom, and 11% reported exchanging sex for money or drugs. One-third of participants reported an incarceration experience during the prior six months. Preliminary

multivariable modeling indicated that factors associated with a higher rate of HCV infection were: sharing injection equipment (adjusted odds ratio (aOR), 2.34, 95% CI 1.34-4.10), history of overdose (aOR, 1.98, 95% CI 1.15-3.41), ever receiving MOUD (aOR=1.98, 95% CI: 1.11-3.51), and being in jail in the past 6 months (aOR, 2.11, 95% CI 1.13-3.94); protective factors included injecting less than daily (aOR, 0.53, 95% CI 0.30-0.93) and having an SSP within walking distance (aOR, 0.53, 95% CI 0.34-0.92).

Mobile syringe service vans can improve syringe access and reduce syringe sharing in rural communities with poor access to SSPs. SSPs provide essential harm reduction and disease prevention services,[24] and serve as bridges to culturally competent care. When SSPs successfully link PWID to treatment through culturally competent providers, overall healthcare utilization among PWID has been positively influenced by trust in their clinical providers. [25-27] Mobile SSPs offer opportunities to meet PWID within local communities, and often attract individuals who are at elevated injection-mediated risk,[28] and who are not served by brick-and-mortar SSP models.[29,30] Research suggests that mobile vans are attractive for those with fewer years of injecting, challenges findings syringes, and incarceration in the previous six months.[28] However, the evidence that SSP access alone can reduce HCV is mixed.[31] We propose that the combination of accessible harm reduction with HCV cure, education and posttreatment surveillance can reduce reinfection.

HCV testing using capillary blood collected on dried blood spot (DBS) cards rather than venous blood can improve the HCV care cascade in rural areas with poor access to phlebotomy. Screening for HCV antibodies can be easily achieved through point-of-care capillary blood testing.[32] To distinguish active from resolved infection, and hence identify persons in need of treatment, a positive antibody test must be followed up with a molecular test detecting viral RNA. Furthermore, in the future, detection of HCV viremia will be essential for HCV screening, since many of the HCV-infected individuals will have been treated.[33] The need for venipuncture to assess HCV viremia leads to a substantial loss to follow up, even in settings with better access to laboratories and less marginalized populations.[34] Indeed, in our UG3 study 26% of individuals with a positive rapid HCV test did not obtain confirmatory testing, largely because of poor venous access. DBS to detect HCV viremia has been implemented internationally in resource poor settings and in hard-to-reach populations, but has not yet been validated in the U.S.[33,35] False negatives from capillary blood collected on DBS cards can occur at low titers of HCV viremia ,[36,37] but patients who experience reinfection usually have high HCV viral loads that are detectable,[38] which will be easily detectable in our UH3 study population.

Adoption of DBS in the US has the potential to improve the HCV care cascade,[39] increase treatment and facilitate monitoring for reinfection in at-risk populations in rural and other resource-poor settings. A major barrier to implementation of DBS is limited experience with its use in different settings. The World Health Organization priorities for research include its validation for monitoring treatment response and HCV test of cure (e.g., SVR12), including threshold for detection, diagnostic performance, and impact on linkage to care in different settings, including mobile and outreach-based testing programs.[40]

Telemedicine can facilitate delivery of HCV treatment to rural areas with a shortage of treating clinicians. Although our original application proposed to adapt elements from two telemedicine models to provide support for local primary care clinicians to care for PWIDs, our work in the UG3 phase and in preparation for the UH3 has found limited willingness and bandwidth of local primary care providers to provide HCV care to persons who are actively injecting drugs. Instead, we propose to provide direct teleconsultation and treatment for HCV among PWIDs in collaboration with local harm reduction agencies. We propose to use a mobile van because of severe space constraints within these agencies, and as a means to facilitate outreach to areas poorly served by brick-and-mortar services. Evidence supports that telehealth treatment for HCV can achieve equivalent or better outcomes than typical referral for face-to-face consultation in rural areas,[41] but significantly reduces the time to a completed consultation from over two weeks to less than one day, with high rates of satisfaction and greater rates of therapy completion.[42,43]

In our UG3 qualitative interviews, nearly all stakeholders viewed telehealth interventions as opportunities to reduce the distances traveled by patients in rural, mountainous, hard-to-reach locations, and increase access to needed services in “treatment deserts”, where there are few Interstates, highways, and a lack of paved

roads. Even in places where Hub and Spoke models have demonstrated great success, it can be challenging for patients to get to their appointments. As two public health officials described:

> …we have folks who really just cannot…access treatment…without taking an hour-long bus ride both ways.

> …it's hard to get around…There's… only…two interstates in a tiny fraction of this state, and most of this state is not covered by major roads…and we have a lot of weather here and a big mountain range in the middle ... And so it can be really hard for people to get access to care, and we don't have a lot of highly trained…specialty providers.

Another salient theme that emerged from our qualitative stakeholder interviews was a lack of infectious disease (ID) trained clinicians. Public health and policy stakeholders could count on two hands the number of ID specialists and gastroenterologists across their respective states, mostly in hospitals and large medical facilities. They noted that "…telemedicine or that type of approach could be really helpful…allowing more providers to reach people…"

HCV treatment can reduce syringe sharing, while reducing the community reservoir of HCV. Studies have shown that treatment for HCV in PWID is associated with a decrease in risk behavior, such as syringe sharing, an increase in medication treatment for opioid use disorder (MOUD) and improved self-care.[44-49] Not only do these changes in injection behavior reduce the likelihood of HCV reinfection, but they protect against HIV transmission. In the context of improved syringe services, our proposed intervention seeks to leverage HCV treatment as a motivator to reduce sharing of injection equipment in order to prevent both HCV reinfection and a Scott County-like outbreak of HIV should it enter local user networks.

Furthermore, treatment as prevention (TasP) is a major component of strategies to combat HIV (identification, linkage, treatment, and retention),[50,51] but has yet to be widely implemented or evaluated for HCV. For the majority of people living with HCV, current DAA treatments could cure them and prevent downstream transmission. Modeling studies suggest that curing a fraction of PWID would decrease prevalent infections, particularly when coupled with other preventive services,[52-54] and may even be cost-saving.[55] These models account for reinfection rates, which are historically relatively low.[56] In other countries TasP is being tested,[57] but the generalizability of international efforts to the U.S. rural population is questionable as contexts, risk behaviors, and reinfection rates may differ substantially. On a population level, it may not be enough to simply offer HCV treatment to PWIDs.[58,59] Opportunities to offer HCV treatment, enhanced access to syringe services, and referral to MOUD in our target study areas may offer the needed combination of services to effectively curb HCV infection rates.

Conceptual Model and Hypotheses. The well-known Gelberg-Andersen Behavioral Model for Vulnerable Populations is the conceptual basis for this study of service delivery to rural PWID.[60] The vulnerable-populations model expands the explanatory domains of predisposing (e.g., age, education), enabling (e.g., health insurance) and need factors (e.g., comorbidities) to include specific vulnerabilities such as substance use, mental illness, and competing needs that are additional barriers to obtaining health care. This model has been shown to be predictive of hepatitis seropositivity and services utilization among homeless adults.[61,62] MPI Friedmann's work has shown that integrative system factors (e.g., on-site care and case management) act as enabling resources that facilitate service delivery among addiction treatment patients.[63-65] Our UH3 telemedicine intervention will be similarly hypothesized to act as an enabling resource that increases access to needed services, and thereby improves outcomes among PWID populations.

Summary of Scientific Premise. This project's significance derives from a critical need to implement and test a comprehensive, integrated and effective model to address the high prevalence of both HCV and syringe sharing among many rural PWIDs as a means to reduce both the community reservoir of HCV and the risk of a Scott Countylike HIV outbreak. Our proposal will compare two strategies to improve access to HCV testing and treatment in rural northern New England counties that our UG3 study has shown to have a high burden of HCV and syringe sharing and limited access to effective services: (1) Enhanced usual care (EUC), in which mobile care provides HCV testing and a care navigator facilitates referral to local or regional clinicians for HCV

treatment; versus (2) Mobile Tele-HCV Care (MTC), in which a mobile van and telemedicine bring HCV testing and treatment directly to the PWIDs' communities. If the mobile approach to HCV testing and treatment and syringe access proves effective, it will provide a promising model for local public health authorities seeking to address immense challenges with opioid injection, syringe sharing and HCV in rural America, and reduce the risk environment for HIV outbreaks in those communities.

IV. Approach.

A. Overall Design.

The proposed UH3 study will employ a randomized, parallel-group design to determine the best strategy for mobile testing-and-treatment of PWIDs for HCV. This Type 1 hybrid effectiveness-implementation study will examine the effectiveness of a model of mobile telemedicine service delivery integrated into rural harm reduction programming.[5] The model aims to reach rural opioid injectors who are living with HCV and to fill service gaps identified in our UG3 study around access to syringe services and HCV testing and treatment. Working closely with local harm reduction agencies, we propose to employ a mobile van to expand antibody and viral load testing for HCV, distribute syringes in rural communities with poor access to brick-and-mortar SSPs, and provide HCV telemedicine treatment to rural PWIDs. We will also validate the accuracy of DBS testing for HCV viral load as a potential strategy to address limited access to effective phlebotomy. All consenting PWIDs with HCV antibody present will receive HCV viral load and liver elastography results, and, if current HCV carriers, initial vaccination for HBV and/or HAV. We will then randomize 220 rural PWIDs with current HCV to one of two strategies for HCV work-up and:

(1) Enhanced Usual Care (EUC) -- referral with care navigation to a local or regional treatment provider.[5]
(2) Mobile tele-HCV Care (MTC) -- telemedicine DAA treatment for HCV on a mobile van.

Primary outcomes will be the proportion of PWID who initiate DAA treatment, achieve sustained virologic response at 12-weeks post-treatment (SVR12), and report no syringe sharing in the prior 30 days at follow-up. We hypothesize that, compared with EUC, mobile tele-HCV care will be associated with:

H1: higher rates of DAA treatment initiation,
H2: higher rates of sustained virological response 12-weeks post-treatment (SVR12, a.k.a. cure), and
H3: lower rates of syringe sharing behavior.

Secondary outcomes will examine rates of completing the HBV and HAV vaccination series, starting MOUD, ED visits, as well as health-related quality of life HRQOL and substance use. Qualitative formative evaluation will assess barriers, facilitators, and implementation lessons-learned.[6]

In order to accomplish the research study aims and to be in compliance with Baystate Medical Center (BMC) policies, the DISCERNNE research team will establish a clinical partnership with Better Life Partners (BLP), who will be responsible for all the following clinical activities and tasks:

1. Patient Registration with BLP;
2. Perform clinical activities, including:
    a. Conduct HCV rapid testing;
    b. Collect Dried Blood Spot (DBS) sample;
    c. Phlebotomy (collect and prepare blood samples for lab analysis);
    d. FibroScan (perform scan, export and send results to study physician).
3. Order, transport, and administer HAV/HBV vaccination.
4. Explain lab results and give referrals for services as appropriate.
5. Assist with ordering HCV medication (for intervention (MTC) group only).
6. Answer clinical/medical questions from participants, as appropriate.

B. Sampling and Data Collection

The study will seek to enroll PWIDs with untreated chronic HCV infection in the 10 rural study counties adjacent to I-91 and the Connecticut River in NH and VT. The following factors will determine study participation:

1. Inclusion Criteria:

a. Current or past history of drug injection;
b. Health insurance that will cover HCV medications (study staff will work with those who are eligible but have not signed up for insurance once consented and enrolled in study)
c. Lives in one of the 10 study counties in NH and VT, and plans to remain in the study region for the next 12 months;
d. Age 18 years or older;
e. Speaks English;
f. Capacity to voluntarily provide informed consent;
g. Will accept randomized assignment, and participate in follow-up over 12 months;
h. Will provide releases to access community medical records;
i. Will provide names and contact information of at least 3 persons for re-contact purposes;
j. Not previously treated for HCV;
k. Not pregnant or trying to conceive;
l. HCV antibody positive on point-of-care rapid test.

2. Exclusion Criteria:

a. Unable to obtain venous blood sample for mandatory laboratory testing
b. HCV viral load undetectable
c. Hepatitis B surface antigen (HBsAg) positive;
d. Significant renal failure (eGFR 30 mL/min/1.73m^2 or less, or end-stage renal disease requiring dialysis);
e. Decompensated cirrhosis, as manifested by liver fibrosis on elastography (through FibroScan) and/or Fibrosure blood test plus at least one of the following symptoms:
    i. Jaundice (yellowing of the eyes and skin)
    ii. Increasing abdominal size (ascites) and leg edema
    iii. Periods of confusion consistent with encephalopathy
    iv. History of gastrointestinal bleeding.

3. Screening and Eligibility.

The 68% of participants recruited during the UG3 phase who tested positive for HCV antibody will be recontacted for screening. We will also screen PWIDs known to be HCV antibody positive who are referred from community partners or eligible participants until we have randomized 220 PWIDs with current HCV infection. Phone or in-person screening by research staff will determine initial eligibility based on self-report of inclusion criteria.

Time T0: Screening. Those who meet these criteria will be invited to the van for *Visit T0,* receive a detailed explanation of the study, undergo informed consent, and receive point of care testing for HCV antibody (if not previously documented) and HIV antibody ***(Figure 1***). If an otherwise eligible participant does not have health insurance, the study staff will assist that person with enrolling in health insurance once consented and enrolled in study.

Persons with HCV antibody present will have phlebotomy performed and undergo van-based non-invasive liver transient elastography; those with high scores for cirrhosis or for whom a successful scan (through Fibroscan) is not able to be completed will have the presence of decompensated cirrhosis confirmed by the FibroSure lab test that will be performed on all HCV RNA positive blood samples. Phlebotomy will be attempted no more than three times in one day for each participant. If an individual attempts a confirmatory blood draw, but a blood sample is not able to be drawn (e.g. phlebotomist is unable to access an appropriate vein after no more than three sticks), the participant may return a second day for no more than three attempts.

If a blood sample is not able to be drawn, the participant would still receive the full $20 payment but would not be eligible to participate if initial lab work cannot be completed. If the confirmatory blood draw is successfully completed, the participant will provide locator information, be paid $20 for their time and discomfort, and asked to revisit the van to receive their results and the final eligibility determination.

Specimens will be dropped at the Baystate Franklin Medical Center laboratory for courier service to Baystate Reference Laboratories (BRL) for determination of HCV viral load (RNA by PCR). For venous samples that are positive for HCV-RNA,BRL will also test for genotype, hepatitis B surface antigen, antibody, and core antibody (HBsAg, HBsAb, and HBcAb). A serum-separating tube (SST) for creatinine, liver function (ALT, AST and Total Bilirubin) and HCG (where appropriate) will be held by BRL. Specimens obtained from individuals that are positive for HCV viremia will be processed for creatinine, liver function and serum HCG tests (where indicated). We anticipate few post-randomization exclusions on the basis of severe renal failure or pregnancy not previously known. Samples positive for HCV viremia will also be sent by BRL to LabCorp for FibroSure testing to confirm the absence of decompensated cirrhosis and will be prepared for eventual batch shipping to the GHOST lab.

Visit T1. Potential participants will return to the van when results have been received.. Study staff will attempt to locate no-show individuals to ensure they receive their laboratory results. Individuals deemed ineligible by laboratory testing will receive a copy of their laboratory results, $10 for their time, a referral for services as appropriate, and an invitation to receive harm reduction services from the van for the duration of the study. Individuals deemed ineligible due to the presence of decompensated cirrhosis as indicated by the FibroSure test will also undergo a tele-exam with a study physician and be referred to a liver specialist. Eligible participants will perform the baseline ACASI interview (for which they will receive $20 for their time), receive the initial vaccination for HBV and HAV as indicated, and be randomized to their study assignment. Participants with a positive test for HIV will be referred to an HIV specialist for HIV care, but HCV treatment will still be provided by the study team according to group assignment.

Randomization (also at Visit T1). Individuals whose specimens are positive for HCV viremia and negative for HBsAg will be randomly assigned 1:1 to Enhanced Usual Care (EUC) or Mobile Tele-HCV Care. Based on feedback gathered during Community Engagement Studios conducted with UG3 study participants, it was determined that randomization should occur in as transparent a manner as possible to address any potential participant concerns around bias or personal discrimination. During Visit T1, eligible participants will sit down with research staff and will witness the randomization process, which will be done using a computer program that utilizes block randomization in real time. Computerized block randomization will ensure balance by study site, gender, and current (last 30 days) drug injection.

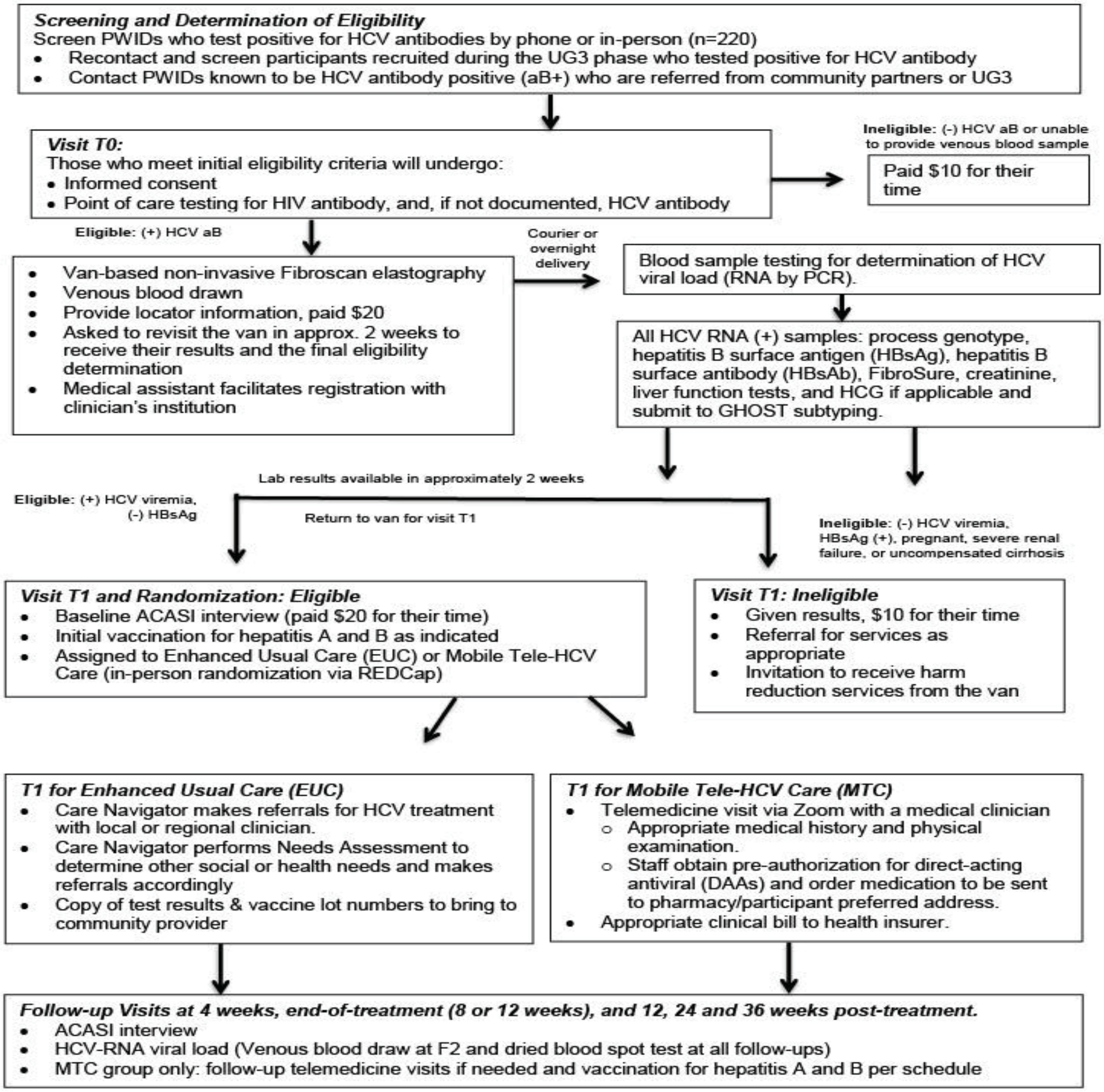

Figure 1. Recruitment and Study flow. Staff on the van will also offer Narcan nasal inhalers, cottons, sterile water, clean cookers, rubber tourniquets, condoms and sterile syringes (provided by harm reduction partners purchased from non-federal sources) at every visit. If resources permit, services would be provided to anyone who approaches the van, but at a minimum, to all who have initiated study procedures (eligible and ineligible).

4. Study Conditions

a. Group 1: Enhanced Usual Care (EUC). At ***Visit T1,*** eligible participants assigned to EUC will meet the care navigator who will arrange referral for HCV treatment with local or regional clinicians, and receive a copy of their test results and information about the initial dose of vaccine they received to bring to the clinician. The care navigation, vaccination, and provision of test results represent significant enhancements to usual care, and may help reduce the high no-show rate for clinical referrals in this population.

Care Navigation. All EUC participants will receive care navigation for HCV treatment. Care navigation will be adapted from existing medical case management services for HIV in consultation with our harm reduction partners. Care navigation will be provided by staff from harm reduction partners and by study staff. Navigation will be tailored to the participant and trauma-informed. The complete manual for HCV care navigation will be

developed in collaboration with harm reduction partners and community stakeholders (including referencing and adapting some of the resources they use for SSP management) in the first quarter of the project.

Care navigation will focus on HCV care coordination and will include maintaining health insurance enrollment,), , coordination of study visits, provision of harm reduction services, and establishment of a care coordination agreement so the care navigator and study team can communicate with providers and other members of the care team. The Care Navigator will also perform an initial assessment of other needs to address social determinants of health and connect participants to available resources, primarily via referrals to harm reduction partners (e.g. MOUD, primary care, visits to the ED, housing, transportation, employment, etc.). EUC participants will have care navigation services for the duration of HCV treatment or, for those who do not initiate treatment, to a maximum duration of 16 weeks.

b. Group 2: Mobile Tele-HCV Care (MTC). At ***Visit T1***, eligible participants assigned to MTC will receive telemedicine visits via Zoom or a similar platform with a medical clinician with the help of the medical assistant or other study staff. Both VT and NH Medicaid cover telemedicine visits without an initial in-person visit. Where possible, DAA treatment will be started at this visit or mailed to the client. The medical assistant or study staff will facilitate consent for treatment, and registration of the patient for the visit with the clinician's institution. An appropriate clinical bill will go to insurance; this process will facilitate sustainability. Follow-up clinical telemedicine visits will be at the discretion of the clinician, with participant meeting the medical assistant for a check-in visit (with study clinician on call, as needed) at week 4 after treatment initiation to monitor initial response and safety, and at end-of-treatment (8 or 12 weeks depending on DAA regimen). Follow-up HBV and HAV vaccines will be scheduled to be given on the van as indicated.

Tele-treatment of HCV with DAAs. Participants in the MTC group will be offered treatment with DAA. In the absence of contra-indications, the pan-genotypic regimen containing glecaprevir-pibrentasvir (Mavyret) will be preferred; the treatment duration is 8 weeks in the absence of cirrhosis, or 12 weeks in the presence of cirrhosis. Patients with mild to moderate renal insufficiency will be offered pan-genotypic sofosbuvir-velpatasvir (Epclusa) for 12 weeks, regardless of the presence of cirrhosis.

5. Data Collection

a. Audio Computer Assisted Survey Instrument (ACASI). Drs. Friedmann and Stopka led development of a harmonized quantitative survey with partners in all eight Rural Opioid Initiative (UG3) sites in 2017-2018. Of the eight UG3 sites, five are currently using the ACASI version developed at the Tufts University School of Medicine. The other three sites are using the harmonized survey in CAPI platforms (RedCap, Qualtrics). Survey domains that are included in all harmonized instruments include: sociodemographics; substance use; injection behavior (including syringe sharing), sex risk behavior, overdose prevalence, substance use treatment, and sexual-mediated risk behaviors; self-reported infectious disease and health status; access to harm reduction (e.g., SSPs, naloxone), MOUD, and health care services; knowledge, attitudes, and experience with such services; geospatial locations of community assets, risks, and place of residence; mobile phone and texting practices and experiences; criminal justice involvement and egocentric and sociometric social networks.

As in the UG3 phase, we will develop and deploy a modified version of our existing ACASI harmonized across the ROI sites to assess participants at baseline and follow-up. We will incorporate domains focused on HCV screening, confirmatory testing, and treatment experiences; telehealth exposure, experience, and satisfaction; additional measures focused on enhanced syringe access, syringe sharing and injection-mediated risks; and experience and satisfaction with our mobile harm reduction and tele-care van. Final core survey questions will be harmonized with those decided upon by the UH3 National Executive Steering Committee. Drs. Friedmann and Stopka are ready and willing to assist the Data Coordinating Center (DCC) with harmonization of measures and instruments across all funded UH3 sites. Where applicable, we will use common data elements such as those from the National Library of Medicine.

b. Follow-up Interval. All participants will have one brief check-in visit approximately 4 weeks after treatment initiation to order more medication or consult with physician (via telemedicine or over the phone, as

needed) if necessary for MTC group, and to check on status of local provider HCV treatment for EUC group. There will then be four follow-up visits: at end-of-treatment (week 8 or 12 after treatment initiation), then approximately 12, 24, and 36 weeks after end-of-treatment to monitor for reinfection. Respondents will complete the ACASI at each follow-up. These assessments will include an HCV-RNA viral load at each follow-up. Once the DBS testing is validated (see below) follow-up viral load testing will no longer utilize venipuncture to determine reinfection. Individuals who do not initiate HCV treatment will have follow-up visits timed as if they had started 8-weeks of treatment 8 weeks after T1. In other words, for those who do not initiate treatment, follow-up will occur approximately 16, 28, 40, and 52 weeks after the T1 visit.

C. Participant Payment

Participants will receive $20 for completion of Screening visit T0. If an individual attempts a confirmatory blood draw at T0, but a blood sample is not able to be drawn (e.g., phlebotomist is unable to access an appropriate vein), the participant may return a second day for no more than three attempts for which they will be paid $20. If a blood sample is not able to be drawn, the participant would still receive the full payment but is not eligible to participate if initial lab work cannot be completed. Participants who are eligible to receive treatment will be asked to complete the ACASI survey at time T1 and will receive $20. Participants deemed ineligible at time T1 will be provided with their results, appropriate referrals and $10. Participants will receive $20 for attending the brief 4 week check-in visit, then $30, $30, $40 and $50, respectively, for completing the procedures at the four follow-up visits.

Enrolled and randomized participants can be paid $20 for successfully referring another eligible participant. In order to receive payment, the referred individual must be fully eligible and randomized; the referring participant will receive payment after the referred participant completes T1.

D. Provision of Syringes, Harm Reduction Kits and naloxone

Study staff will distribute sterile syringes to and collect used syringes from PWIDs in keeping with community standards. Syringes will be provided by our harm reduction partners and will not be purchased with federal funds. We will prepare and distribute harm reduction kits (e.g. condoms and sterile tourniquets, cottons, cookers and waters), and information on local treatment resources to interested participants. As in the UG3 phase, study staff will receive training on how to use and distribute naloxone. The states of Vermont and New Hampshire have agreed to supply study staff with naloxone for distribution.

E. Analysis Plan.

Primary outcomes will be the proportion of PWID who initiate DAA treatment, achieve sustained virologic response at 12-weeks post-treatment (SVR12), and report no syringe sharing in the prior 30 days at follow-up. We hypothesize that, compared with EUC, mobile tele-HCV care will be associated with:

H1: higher rates of DAA treatment initiation,
H2: higher rates of sustained virological response 12-weeks post treatment (SVR12 or cure), and
H3: lower rates of syringe sharing behavior among rural HCV-infected PWIDs

Secondary outcomes will include HBV and HAV vaccination series completion rates, initiation of MOUD and ED visits, as well as health-related quality of life (HRQOL) and substance use.

Preliminary Analyses: Initial data analysis will describe the distribution of all outcomes by study group. Continuous measures will be reported using means, standard deviations, medians and percentiles. Categorical measures will be reported using frequencies and percentages. Rather than testing for baseline covariate imbalance, stratified analyses will be conducted for baseline covariates that are known to be strongly associated with outcomes, as well as those that show imbalance on inspection.[70,71]

Primary outcome analyses. We will apply ICH-E9 guidelines[72,73] for statistical analysis and the CONSORT statement[74] for reporting of findings. Since Aims 1 and 2 examine the effectiveness of the intervention by comparing proportions in the two study groups, we will apply the same statistical approach. For the univariable approach, study groups will be compared using Pearson's chi-square or Fisher's exact test. Stratification and adjustment for relevant baseline covariables will be accomplished with logistic regression. In

these models, study group and other categorical measures will be represented with indicator variables. Continuous explanatory variables will be evaluated to verify linearity of their logit and, if not, they will be rescaled. Model fit will be examined and final results will be expressed as odds ratios and adjusted proportions each with 95% confidence intervals. Significance testing will be conducted at a critical level of 5%.

Heterogeneity of Treatment Effect. Heterogeneity of treatment effect will be examined by both pre-planned sub-group analyses and more exploratory approaches.[75,76]. Pre-specified sub-groups will be defined based on a) demographic factors, such as age, race/ethnicity, income (if available), marital status, and b) opioid use characteristics, such as duration of use. We will follow best practice and base tests of statistical significance on regression-based interaction terms (e.g., interaction of sub-group status with study group) so that the full sample size is used to maximize power. Further, to address the loss of power with sub-group analyses, we will explore for clinical relevance any planned interaction achieving p≤0.20. More exploratory approaches will be used to classify participants according to the likelihood of intervention using regression-based approaches, potentially yielding a layer of insight not available from the more restrictive pre-defined sub-group analyses.

Missing Data. Missing data may arise from subject dropout, non-compliance, or item nonresponse. Several approaches to assess the impact will be undertaken. In general, we will use all available data regardless of whether a patient has a complete record. Participants with missing data will be compared to those without to check for differences. We will explore missing data to evaluate the presence of patterns. Secondly, making the assumption that the data are missing at random (MAR), multiple imputation methods will be used to generate data for the missing values based upon the distribution of available data (using SAS PROC MI or Stata's MI IMPUTE), and results of multiple sets combined into a summary measure with appropriate statistical tests (SAS PROC MIANALYZE or Stata's MI ESTIMATE). Results from each strategy will be compared to analyses using the complete data.

Sample size. Sample size and power estimates are based on a comparison of two proportions (PASS v.15). From the UG3 phase of the study, we enrolled 589 participants, of which approximately 344 tested positive for HCV. Among these with supplementation, we will recruit at least 220 (about 64%) for the UH3 phase. Because mobile vans are expensive to implement, we believe that, at a minimum, a moderate effect size should be required to be meaningful from the public health, clinical and policy perspectives.

For treatment initiation, we expect 50% or fewer participants in the EUC to initiate treatment, and the MTC intervention to improve initiation to at least 70%. Thus, 220 individuals randomized equally to two groups would provide 86% power to detect a 20% absolute difference between study groups for a test of difference in proportions at P<.05. This difference is equivalent to an odds ratio of 2.33, or a medium effect size.[77]

For sustained viral response at 12 weeks post-treatment (SVR12), an intent-to-treat approach would include all participants randomized. We expect the success rates to be lower than for the "as-treated" approach, since attrition may occur both at initiation of therapy, as well as completion of therapy. Thus a sample of 110 participants per group would provide about 80% power to detect difference in the proportion of treatment success of 30% in the EUC and 48.5% in the MTC group. This difference is equivalent to an odds ratio of 2.12, which is equivalent to a medium effect size (approximate Cohen's d = 0.42). Because the lower treatment initiation rate in the EUC group will favor the MTC group, we also present an as-treated sample size calculation. We expect about 55 patients (i.e., 50% of 110) in the EUC group and at least 77 patients (70% of 110) in the MTC to initiate treatment. Again, we expect the MTC intervention to overcome substantial obstacles to achieving SVR12. As such, a difference in the proportion of participants achieving SVR12 at least as large as 25% is expected. Thus a sample of 132 participants (55 in usual care and 77 in intervention), would provide about 82% power to detect a difference between the MTC intervention and EUC groups of 25% absolute difference (i.e., 65% vs. 40%) for a test of difference in proportions at a critical level of 5%. This difference is equivalent to an odds ratio of 2.79, which is about a medium effect size (equivalent to Cohen's d of 0.56)

For injection sharing behavior, our UG3 survey found that 77% of HCV-infected PWIDs shared *any* equipment in the 30 days prior, and 66% shared syringes. Using an ITT approach, a sample of 110 participants per group would provide at least 80% power to detect a 19% or greater reduction in sharing behaviors for

either measure (i.e., 77% vs. 58% for sharing any equipment, and 66% vs. 47% for sharing syringes only) for a two-sided test in the difference in proportions at a critical level of 5%. The 19-point difference for these outcomes yields an OR of about 2.4, which equates to a medium effect size (approximate Cohen's d = 0.48).

F. Determination of SVR12 and reinfection by dried blood spot (DBS) specimens

To determine SVR12, we will compare the sensitivity and specificity of HCV RNA detection by PCR from a venous blood sample collected in standard blood collection tubes with a capillary blood sample collected on DBS. We will use the DBS alone to screen for reinfection two more times, every 12 weeks after SVR12 through the end of the study period.

Twelve weeks after treatment completion, we will collect a venous sample to be processed at the Baystate Laboratory on their routine, clinical platform to detect HCV RNA by PCR. An undetectable RNA at this time point would establish SVR12, also considered cure. A simultaneously collected capillary sample will be processed Molecular Testing Labs (MTL) in Vancouver, WA, according to their validated procedure for DBS processing and detection of HCV RNA by PCR. We will establish sensitivity and specificity of the DBS method, using the detection of HCV RNA by PCR from the venous sample as the gold standard.

After SVR12 has been established, we will collect capillary blood on DBS two more times, every twelve weeks, to be processed by MTL, to screen for reinfection.

G. Qualitative Formative Evaluation

Systematic Observation and Evaluative Ethnography. MPI Stopka will lead the systematic observation of the UH3 intervention in action and evaluative ethnography capturing the complexity of the overall syringe services and HCV telehealth implementation process within the larger harm reduction context, on and around the van.[6] We will conduct a series of observation episodes and take detailed ethnographic field notes at selected sites where the UH3 prevention and treatment van provides services. Observations and ethnographic field notes will be directed by a guide that will aim to capture the following:

1) Description of the harm reduction and HCV care continuum setting (nature of setting, time of day, month, spatial arrangements of people in the settings);
2) Details on how syringe services and HCV telehealth are used, including implementation, ease of use, staff interactions, experiences and reflections;
3) Description of syringe service and HCV telehealth staff (services offered, skills, rapport with clients);
4) Description of clients utilizing syringe service and HCV telehealth, their sociodemographic characteristics (e.g., sex, race/ethnicity, age), drugs of choice, and unique needs;
5) The interactions between staff and clients (type of interaction, casual conversation topics, disposition of staff toward clients and vice versa, services provided).

We will record all observations that appear to be pertinent to our core programmatic evaluation. From what we learn from these observations, we will develop a list of questions to ask in the form of "on the spot interviews" with service providers and clients. We will focus on the following guiding topics: a) implementation; b) acceptability; c) feasibility; d) participant perceptions; e) staff, provider, and partner perceptions at UH3 mobile sites and local harm reduction and public health agencies; f) barriers, facilitators, successes, challenges and lessons-learned; g) larger relationships between the syringe service and HCV telehealth van and the harm reduction and HCV care continuums; and, h) sustainability. We will ask questions focused on these topics whenever possible, before or immediately after the encounter between service providers and clients. Recorded data from these ethnographic observations, conversations, and information elicited from "on the spot interviews" will be transcribed and entered into a word processing software as textual/content data for subsequent analysis.

H. Qualitative Data Analysis. Our team will conduct qualitative analyses in two stages: open coding, and re-coding by application of codes from the coding manual developed in the study. Through secondary coding, we will group first-generation codes into larger themes. We will enter coded text and coding tree into Dedoose v8, which will allow us to group segments from all documents by code in a secure cloud-based system that is ideal

for cross-site coding and analysis. By creating a database that can be indexed by topic or theme, we will be able to identify patterns, clusters, and relationships across all cases in the data, such as experiences with syringe services and HCV telehealth, perceived benefits and challenges with services among stakeholders, and knowledge, attitudes and beliefs about van-based syringe services and HCV treatment among PWIDs.

We will enter preliminary ideas for codes in Dedoose following initial readings of transcripts. This process will continue until the conceptual categories are exhausted or when few new ideas emerge that have not already been noted (i.e., when we achieve thematic saturation). We expect to reach saturation following approximately 75 interviews including study staff working on/around the van, harm reduction and public health stakeholders, telemedicine and community healthcare providers and study participants. Next, our DISCERNNE qualitative research team members will refine and reduce the number of open codes by comparing and contrasting them and defining the limits or boundaries of particular conceptual categories. This process will continue iteratively until acceptable inter-coder reliability has been achieved. The remaining transcripts will then be divided among the coding team members and independently coded by the five team members as we have done in the UG3 phase. The coding team will continue to meet regularly via videoconference (e.g., Zoom) to resolve coding questions. Once the coding manual is final and data are coded, we will begin the analytic process. We will use thematic analysis to identify salient patterns in the data with regard to harm reduction and HCV treatment care continuum experiences on the van. In addition to providing an improved understanding of the intervention elements that facilitate or impede implementation, the formative evaluation will be used to optimize the intervention during the remaining time in the project.

IV. DATA AND SAFETY MONITORING

The conduct of all human studies research carried out during the UH3 phase will follow guidelines established by the National Executive Steering Committee, NIDA, and local IRBs. Research subjects for the proposed project will include key informant public health stakeholders (i.e., local/regional clinicians), Persons Who Inject Drugs (PWID), study staff (i.e., staff working on/around the van; telemedicine clinicians), and the staff of harm reduction agencies in rural counties in the states of Vermont and New Hampshire.

A. Statement of responsibility.

The mPIs are responsible for data safety and monitoring for the project, as well as developing and executing a Data & Safety Monitoring Plan (DSMP), which will be reviewed and monitored by the NIDA Program Officer, the Baystate IRB and a Data Safety and Monitoring Board (DSMB). The DSMP will be completed and approved by the Program Officer, the Baystate IRB and DSMB before data collection commences. The study will monitor adverse effects and collect information that will allow the intervention to be used safely.

B. DSMB plan.

A DSMB will be established to monitor the safety of the subjects, the study/data validity, the effectiveness of the medications under study, and any unexpected effects. The DSMB will ensure immediate and appropriate response to any sign of harmful effects with the potential to justify stopping study activities.

Composition. We propose a 3-person DSMB that includes a physician expert in addictions, an epidemiologist or biostatistician, and an individual representing the interests of the affected population. They will have neither professional nor financial interests in the study outcome, and will not be employed by the funding agency. Each member will bring expertise in the varied scientific disciplines needed to interpret the data and ensure participant safety. Resumes will be made available to the Program Officer and IRB.

Charge. The DSMB is charged with ensuring the safety of individuals participating in this study. Its activities will include reviewing any AEs or SAEs that occur, approving and overseeing the plan for interim data analysis,

and preparing written reports to inform the mPIs, as well as the Program Officer, of the impact of the research on the safety of the participants.

Frequency of DSMB reviews. The DSMB will meet via videoconference at the discretion of its Chair, but at a minimum 3 times – (1) prior to the commencement of data collection to review (and revise as necessary) the detailed DSMP (which will have been approved by the Program Officer and IRB), and ensure that a clear protocol for study monitoring is operational prior to data collection activities, (2) after the first 25% of subjects completes end-of-treatment assessment, and (3) after the first 50% subjects completes 12-week follow-up.

Content of DSMB report. The DSMB will send a report to the NIDA Program Officer within a month after each DSMB meeting, which will include meeting dates, meeting agenda and minutes, changes in membership (including qualifications and conflicts of interest for any new members), and DSMB recommendations for the study.

C. Staff training.

All research-engaged staff will be required to have completed CITI human subjects and Good Clinical Practice trainings, as well as training in study procedures. New research staff are required to observe the consent procedure for at least 3 subjects and have at least three of their initial attempts at the consent process observed by the research coordinator or an investigator before consenting independently. The Baystate Human Research Protection Program (HRPP) provides 1-on-1 training to orient new staff to working in research and has developed a competency-based manual for training research staff in proficiencies needed to function in clinical research roles. The HRPP training includes the following domains: Scientific Concepts and Research Design; Ethical and Participant Safety Concerns; Medicine Development and Regulation; Study and Site Management and Data Management and Informatics.

D. Data Management and Analysis

1. Data Acquisition, transmission, management and storage. The study will involve audio-assisted computer-based interviews, and blood tests. We will deploy a modified version of the existing ACASI harmonized across the Rural Opioid Initiative UG3 sites to assess participants at baseline and follow-up**.** We will incorporate domains focused on HCV screening, confirmatory testing, and treatment experiences; telehealth exposure, experience, and satisfaction; additional measures focused on enhanced syringe access, sharing and injection-mediated risks; and experience and satisfaction with our mobile harm reduction and tele-care van. Final core survey questions will be harmonized with those decided upon by the UH3 National Executive Steering Committee. Survey domains that are already present include: sociodemographics; substance use; , injection behavior (including syringe sharing), sex risk behavior, overdose prevalence, substance use treatment, - and sexual-mediated risk behaviors; self-reported infectious disease and health status; access to harm reduction (e.g., SSPs, naloxone), medication for OUD (MOUD), and health care services; knowledge, attitudes, and experience with such services; geospatial locations of community assets, risks, and place of residence; mobile phone and texting practices and experiences; criminal justice involvement and egocentric and sociometric social networks. As in our UG3 project, the computer-based interviews will be conducted using a password-protected application on an encrypted, password-protected laptop computer in a private space with the participant wearing headphones.

All participants will have up to four follow-up visits at end-of-treatment (week 8 or 12 after treatment initiation), then 12, 24, and 36 weeks after end-of-treatment to monitor for reinfection. Respondents will complete the ACASI at each follow-up. These assessments will include an HCV-RNA viral load at each follow-up. If the dried blood spot testing is validated follow-up viral load testing will no longer utilize venipuncture to determine reinfection. Individuals who do not initiate HCV treatment will have follow-up visits timed as if they had started 8-weeks of treatment 8 weeks after T1. In other words, for those who do not initiate treatment, follow-up will occur 16, 28, 40, and 52 weeks after the T1 visit. Participants will receive $30, $30, $40 and $50, respectively, for completing the procedures at the four follow-up visits.

Transmission procedures will adhere to those specified by the Data Coordinating Center and the ESC. Analytic datasets will be stripped of patient identifiers, with re-identifying crosswalks kept in a separate, secure place. To protect against inappropriate access, we will first handle and transmit only de-identified data wherever possible. Data transmissions will be handled over secure VPN connections or other secure procedures, and data will be encrypted wherever feasible. Data forms and other records will be located in a secure environment with access restricted to authorized personnel, with backup copies kept in a separate, secure location.

Data management and storage must be secured against loss and inappropriate access. We will implement best-practice data collection and handling procedures, with well-documented procedures, and training and monitoring protocols that assure compliance with evolving federal and industry standards to protect identifying information and prevent breaches of confidentiality. Identifiers will be stripped and kept separate from the data in locked files available to only authorized staff; computers are encrypted and password-protected, and databases are password-protected as well. Electronic data back-ups will be performed at least daily. Data services will be provided by personnel located in the Epidemiology and Biostatistical Research Core at Baystate Medical Center.

2. Data entry methods. The study will involve audio-assisted computer-based interviews. All other data, including monitoring and scheduling data, will be entered through a secure, internet-enabled implementation of REDCap (Research Electronic Data Capture, Vanderbilt University). During recruitment and data collection, the database will be used to maintain contact rosters and provide prompts for multiple contacts. Multiple data views will be available to monitor recruitment and data completeness, so that problems can be pinpointed early and preventive action taken. Within the REDCap database, we will include date and time stamp fields for study process measures. During regularly scheduled staff meetings, we will review data on the process measures to

insure expected contacts and response times are met. Monitoring process measures will also enable us to identify and correct early any deviations in our enrollment and follow-up activities.

E. Quality Assurance & Regulatory Issues

1. Quality assurance plan.

Data Management will adhere to the highest standards of data quality and confidentiality.

Data cleaning. Mr. Hoskinson, Ms. Wilson and Dr. Visintainer will ensure that all data are validated for completeness and consistency using checks for range and logical consistency. We will also work with the collaborative in data checking, cleaning, transmission and/or analysis; responding to inquiries from the ESC, the Data Coordinating Center (DCC) and Data Safety Monitoring Board (DSMB), and participating sites; compiling data for interim analyses as needed, and sending findings to appropriate entities. At regularly scheduled intervals defined by the ESC, study progress will reported to the ESC and DCC; the DSMB will receive data and reports quarterly. Our group uses REDCap for data management, and SAS and STATA as statistical packages; a REDCap-based tracking system also manages follow-up data collection and monitors data receipt.

Missing data may arise from subject dropout, non-compliance, or item nonresponse. Several approaches to assess the impact will be undertaken. In general, we will use all available data regardless of whether a patient has a complete record. Participants with missing data will be compared to those without to check for differences. We will explore missing data to evaluate the presence of patterns. Secondly, making the assumption that the data are missing at random (MAR), multiple imputation methods will be used to generate data for the missing values based upon the distribution of available data (using, such as, SAS PROC MI or Stata's MI IMPUTE), and results of multiple sets combined into a summary measure with appropriate statistical tests (SAS PROC MIANALYZE or Stata's MI ESTIMATE). Results from each strategy will be compared to analyses using the complete data.

2. Reporting mechanisms and timeless for reporting of AEs/SAEs.

An AE is defined as any unexpected reaction, side effect, or untoward event that occurs during the course of the trial. Staff will assess AEs by asking the following question at each contact: "Since we last met have you had any physical, emotional, mental, or behavioral problems or situations arise in your life that were problematic for you?" Stable chronic conditions that are present prior to study entry and do not worsen are not considered AEs. AEs and SAEs will be identified by a *proactive system of incident reports*, which are reviewed by the mPIs and staff, who will assign a classification to represent the event's documentation as an SAE and whether there is attribution to the study intervention or research procedures.

The mPIs are responsible for ensuring that the Program Officer, IRB, and DSMB receive regular reports of SAEs, AEs, other unanticipated problems, and any modifications to study procedures or protocols. Drs. Friedmann and Stopka will ensure that all research staff are properly trained in the assessment of SAEs and AEs, and that a robust system of recording and reporting SAEs and AEs is in place and fully understood by research staff. The definitions of SAEs and AEs and procedures for reporting such events will be routinely covered during staff training; review and reinforcement of this information will be part of regular supervisory meetings, and AEs are a regular agenda item for project meetings. Research assistants will inquire about SAEs and AEs at each research interview, and will incorporate self-report to ensure timely identification of SAEs and AEs.

Adverse events will be an agenda item at project meetings at least monthly. Any adverse event that comes to the attention of the mPIs or the Project Director will be reported to the Baystate IRB, which will serve as the single IRB. Depending on the nature and seriousness of the adverse event, either the study protocol or the informed consent form may be changed, with the advice and approval of the Baystate IRB and NIDA. AEs will be reported to the NIDA PO at least once per year as a part of the annual progress report. At a minimum, this report will describe the event, when it occurred, the study arm of the participant, and the outcome/resolution. If

there were no AEs, a statement that no AEs occurred will be included in the progress report or communicated to the PO in writing.

All SAEs will be reported within 24 hours to the NIDA Program Officer, IRB and DSMB by email. This 24-hour notification will include a brief explanation of the SAE and when it occurred. A written follow up report will be sent within 72 hours of the event. The written follow up will include information on the date of the event, what occurred, actions taken by project staff, planned follow up (if any), the intervention group/study arm of the affected participant, whether the event appears to be related to the intervention, and whether participant will continue in the study). Based on the report, the DSMB will determine if sufficient evidence exists to qualify an SAE as requiring either further review, modification of the protocol, or suspension of data collection; it will issue such a report within 3 days of notification. The DSMB and IRB have the authority to suspend or terminate the research when the study intervention causes unexpected serious harm to participants. Resumption of data collection activities is contingent on resolution of the sources of the event and determination that the safety of participants will be assured if project resumes; concurrence among the DSMB, IRBs, Program Officers, Principal Investigator and local investigators will be communicated to NIDA. Changes that may need to be made to the protocol will be discussed with the NIDA Program Officer in advance. Agreed upon changes will be submitted as an amendment to the IRB and DSMB for approval. The mPIs will report such changes in the annual progress report to NIDA, along with analysis of cumulative SAEs and AEs to date.

4. *Potential risk and benefits for participants*.

For this study, AEs will include symptoms reported by the patient and abnormal measures of clinical importance noted by study staff. AEs will also include breaches of confidentiality; reports of current and/or intended physical harm to persons (including current and/or intended abuse of children or elders, or an investigation of such allegations(s)); as well as reports of discomfort from or dissatisfaction with assessment procedures, embarrassment in disclosing sensitive personal information, or dissatisfaction with intervention activities. Each AE will be classified by the study investigators as serious or non-serious and appropriate reporting procedures followed. For purposes of this data safety plan, SAEs are defined as any fatal event, any immediately life-threatening event, any permanent or substantially disabling event, and any event that results in hospitalization. Overall, direct-acting antiviral (DAA) regimens are extremely well tolerated, with only mild to moderate side effects. The most common adverse effects include headache, fatigue, diarrhea, nausea, pharyngitis, and insomnia, with fewer than 1% of patients in clinical trials stopping the medication because of adverse effects. Reactivation of hepatitis B virus (HBV) infection has been described during DAA treatment, thus this study will screen and exclude persons positive for HBsAg. Other than these risks associated with standard clinical care for HCV infection, the potential additional risk to study participants is minimal and includes breach of confidentiality and the discomfort of fingersticks and venipuncture. The benefits to participants include improved access to HCV virologic testing and effective treatment.

***5. Plans for interim analysis*.**

At least quarterly during one of its weekly meetings, the Executive Committee will review AE and SAE reports for safety issues, recruitment attainment (including participants screened, impact of eligibility criteria, refusal, and expected accrual rate), balance of key characteristics on randomization, follow-up adequacy (including follow-up rates and on-time rates (within 2 weeks), and missing data on key variables (including HCV viral load). This information will be compiled and shared with the DSMB at its periodic meetings. One formal interim analysis for efficacy is planned for when half of enrolled participants have been evaluated for sustained virologic response at 12-weeks post-treatment (SVR12).

6. Trial stopping rule.

Early termination for efficacy is based on the difference between study groups on the proportion achieving SVR12. It is computed using the O'Brien-Fleming estimate of the alpha-spending function for one interim (at

50% at 12-weeks post-treatment) and one final test of significance. At the interim look, if the difference in proportion of completing treatment achieves of p-value of 0.003 or less, enrollment will stop due to efficacy. If the study continues to completion, the final p-value for testing adjusted for the interim look will be 0.047. At the interim look, we will also consider stopping for futility. This decision will be based on conditional power (Proschan, Lan, Wittes, 2010). Using the interim data, conditional power will be computed under two conditions: a) conditional power under the originally-hypothesized estimates, and b) conditional power under the observed data. If both estimates of conditional power fall below 0.10, then then we consider stopping the study for futility. While a low conditional power will inform the decision to stop the study, the final decision to terminate the trial will consider other factors, such as participant engagement and care delivery.

7. Conflict of interest.

The researchers involved in this study do not have any known conflict of interest issues in this Data and Safety Monitoring Plan. The IRB will verify that no conflicts of interest exist as part of their review. Given the nature of the study, it is unlikely that an unmanageable conflict of interest could arise. If a conflict of interest emerges that cannot be rectified, the conflicted staff will be reassigned. DSMB members will not include anyone with a conflict of interest.

V. CLINICALTRIALS.GOV REQUIREMENTS

The UH3 randomized, open-label, parallel-group study will be registered in ClinicalTrials.gov.

VI. REFERENCES

1. Rudd RA, Aleshire N, Zibbell JE, Gladden RM. Increases in Drug and Opioid Overdose Deaths--United States, 2000-2014. *MMWR Morb Mortal Wkly Rep.* Jan 01 2016;64(50-51):1378-1382.
2. Conrad C, Bradley HM, Broz D, et al. Community Outbreak of HIV Infection Linked to Injection Drug Use of Oxymorphone--Indiana, 2015. *MMWR Morb Mortal Wkly Rep.* May 2015;64(16):443-444.
3. Rudd RA, Seth P, David F, Scholl L. Increases in Drug and Opioid-Involved Overdose Deaths — United States, 2010–2015. *MMWR Morb Mortal Wkly Rep.* 2016. http://dx.doi.org/10.15585/mmwr.mm655051e1.
4. Thakarar K, Rokas K, Lucas FL, et al. Health Disparities, Mortality and Morbidity: Epidemiological Patterns in the Setting of the Opioid Epidemic. Paper presented at: Maine CDC Infectious Disease Conference, November 20162016; Augusta, ME.
5. Curran GM, Bauer M, Mittman B, Pyne JM, Stetler C. Effectiveness-implementation hybrid designs: combining elements of clinical effectiveness and implementation research to enhance public health impact. *Med Care.* Mar 2012;50(3):217-226.
6. Stetler CB, Legro MW, Wallace CM, et al. The role of formative evaluation in implementation research and the QUERI experience. *J Gen Intern Med.* Feb 2006;21 Suppl 2:S1-8.
7. Van Handel M. County-Level Vulnerability Assessment for Rapid Dissemination of HIV or HCV Infections Among Persons Who Inject Drugs, United States. *J Acquir Immune Defic Syndr.* 2016;73.
8. Kolodny A, Courtwright DT, Hwang CS, et al. The prescription opioid and heroin crisis: a public health approach to an epidemic of addiction. *Annu Rev Public Health.* Mar 2015;36:559-574.
9. Martin M, Vanichseni S, Suntharasamai P, et al. Risk behaviors and risk factors for HIV infection among participants in the Bangkok tenofovir study, an HIV pre-exposure prophylaxis trial among people who inject drugs. *Plos One.* 2014;9(3):e92809-e92809.
10. Broz D, Wejnert C, Pham HT, et al. HIV infection and risk, prevention, and testing behaviors among injecting drug users -- National HIV Behavioral Surveillance System, 20 U.S. cities, 2009. *MMWR Morb Mortal Wkly Rep. Surveillance Summaries (Washington, D.C.: 2002).* 2014;63(6):1-51.
11. Prevention CfDCa. Integrated prevention services for HIV infection, viral hepatitis, sexually transmitted diseases, and tuberculosis for persons who use drugs illicitly: summary guidance from CDC and the U.S.

Department of Health and Human Services. *Recommendations and reports : MMWR Morb Mortal Wkly Rep. Recommendations and reports.* Nov 09 2012;61:1-40.
12. Zibbell JE, Iqbal K, Patel RC, et al. Increases in hepatitis C virus infection related to injection drug use among persons aged </=30 years - Kentucky, Tennessee, Virginia, and West Virginia, 2006-2012. *MMWR Morb Mortal Wkly Rep.* May 08 2015;64(17):453-458.
13. Johnson NB, Hayes LD, Brown K, et al. CDC National Health Report: leading causes of morbidity and mortality and associated behavioral risk and protective factors--United States, 2005-2013. *MMWR Morb Mortal Wkly Rep. supplements.* Oct 31 2014;63:3-27.
14. Ly KN, Xing J, Klevens RM, Jiles RB, Ward JW, Holmberg SD. The increasing burden of mortality from viral hepatitis in the United States between 1999 and 2007. *Ann Intern Med.* 2012;156:271-278.
15. Center for Disease Control and Prevention. Diagnoses of HIV infection in the United States and dependent areas, 2017. In: Report HS, ed. Vol 292018.
16. Center for Disease Control and Prevention. HIV infection, risk, prevention, and testing behaviors among persons who inject drugs-National HIV behavioral surveillance: Injection drug use, 20 U.S. Cities In: Report HSS, ed. 2012.
17. Broz D, Zibbell J, Foote C, et al. Multiple injections per injection episode: High-risk injection practice among people who injected pills during the 2015 HIV outbreak in Indiana. *Int J Drug Policy.* 02 2018;52:97-101.
18. Cranston K, Alpren C, John B, et al. Notes from the Field: HIV Diagnoses Among Persons Who Inject Drugs - Northeastern Massachusetts, 2015-2018. *MMWR Morb Mortal Wkly Rep.* Mar 2019;68(10):253-254.
19. Schumaker E. Opioids Have Sparked an HIV Epidemic in Massachusetts. 2018; https://www.huffingtonpost.com/entry/massachusetts-fentanyl-opioids-hiv_us_5b6470bfe4b0b15abaa2958c. Accessed August 6, 2018.
20. Health MDoP. HIV treatment guidelines and clinical advisories. 2019; https://www.mass.gov/lists/hiv-treatment-guidelines-and-clinical-advisories. Accessed April 29, 2019.
21. Suryaprasad AG, White JZ, Xu F, et al. Emerging epidemic of hepatitis C virus infections among young nonurban persons who inject drugs in the United States, 2006-2012. *Clin Infect Dis.* 2014;59(10):1411-1419.
22. Center for Disease Control and Prevention. Vermont - 2015 State Health Profile. 2016. https://www.cdc.gov/nchhstp/stateprofiles/pdf/vermont_profile.pdf. Accessed 12/18/2016.
23. Center for Disease Control and Prevention. Viral Hepatitis Surveillance - United States, 2014. 2016. https://www.cdc.gov/hepatitis/statistics/2014surveillance/pdfs/2014hepsurveillancerpt.pdf.
24. Wodak A, Cooney A. Do needle syringe programs reduce HIV infection among injecting drug users: a comprehensive review of the international evidence. *Subst Use Misuse.* 2006;41(6-7):777-813.
25. Guerrero EG. Enhancing access and retention in substance abuse treatment: the role of Medicaid payment acceptance and cultural competence. *Drug Alcohol Depend.* Oct 2013;132(3):555-561.
26. Mojtabai R, Chen LY, Kaufmann CN, Crum RM. Comparing barriers to mental health treatment and substance use disorder treatment among individuals with comorbid major depression and substance use disorders. *J Subst Abuse Treat.* Feb 2014;46(2):268-273.
27. Ostertag S, Wright BRE, Broadhead RS, Altice FL. Trust and other Characteristics Associated with Health Care Utilization by Injection Drug Users. *J Drug Issues.* 2006;36(4):953-974.
28. Miller CL, Tyndall M, Spittal P, Li K, Palepu A, Schechter MT. Risk-taking behaviors among injecting drug users who obtain syringes from pharmacies, fixed sites, and mobile van needle exchanges. *J Urban Health.* Jun 2002;79(2):257-265.
29. Strike C, Miskovic M. Scoping out the literature on mobile needle and syringe programs-review of service delivery and client characteristics, operation, utilization, referrals, and impact. *Harm Reduct J.* 02 2018;15(1):6.

30. Islam MM, Conigrave KM. Assessing the role of syringe dispensing machines and mobile van outlets in reaching hard-to-reach and high-risk groups of injecting drug users (IDUs): a review. *Harm Reduct J.* 2007;4:14.
31. Davis SM, Daily S, Kristjansson AL, et al. Needle exchange programs for the prevention of hepatitis C virus infection in people who inject drugs: a systematic review with meta-analysis. *Harm Red J.* 2017;14(1):25.
32. Chevaliez S, Poiteau L, Rosa I, et al. Prospective assessment of rapid diagnostic tests for the detection of antibodies to hepatitis C virus, a tool for improving access to care. *Clin Microbiol Infect.* 2016;22(5):459 e451-456.
33. Vazquez-Moron S, Ryan P, Ardizone-Jimenez B, et al. Evaluation of dried blood spot samples for screening of hepatitis C and human immunodeficiency virus in a real-world setting. *Scientific Rep.* 2018;8(1):1858.
34. McGibbon E, Bornschlegel K, Balter S. Half a diagnosis: gap in confirming infection among hepatitis C antibody-positive patients. *Am J Med.* 2013;126(8):718-722.
35. Nguyen TT, Lemee V, Bollore K, et al. Confirmation of HCV viremia using HCV RNA and core antigen testing on dried blood spot in HIV infected peoples who inject drugs in Vietnam. *BMC infectious diseases.* 2018;18(1):622.
36. Lange B, Roberts T, Cohn J, et al. Diagnostic accuracy of detection and quantification of HBV-DNA and HCV-RNA using dried blood spot (DBS) samples - a systematic review and meta-analysis. *BMC infectious Dis.* 2017;17(Suppl 1):693.
37. Soulier A, Poiteau L, Rosa I, et al. Dried blood spots: a tool to ensure broad access to hepatitis c screening, diagnosis, and treatment monitoring. *J Infect Dis.* 2016;213(7):1087-1095.
38. Greenman J, Roberts T, Cohn J, Messac L. Dried blood spot in the genotyping, quantification and storage of HCV RNA: a systematic literature review. *J Viral Hepatitis.* 2015;22(4):353-361.
39. Bregenzer A, Conen A, Knuchel J, et al. Management of hepatitis C in decentralised versus centralised drug substitution programmes and minimally invasive point-of-care tests to close gaps in the HCV cascade. *Swiss Med Wkly.* 2017;147:w14544.
40. WHO. Guidelines on hepatitis B and C testing. *Licence: CC BY-NC-SA 3.0 IGO.* 2017; https://www.who.int/hepatitis/publications/guidelines-hepatitis-c-b-testing/en/. Accessed May 1, 2019.
41. De Gijsel D, Kruger B, Hakim D, Moore S. Telemedicine for the Treatment of Hepatitis C: A Systematic Review and Meta-Analysis. *Open Forum Infectious Dis.* 2018;5(Suppl 1):S31. https://doi.org/10.1093/ofid/ofy209.071.
42. Totten AM, Hansen RN, Wagner J, al. e. Telehealth for Acute and Chronic Care Consultations. Comparative Effectiveness Review No. 216 (Prepared by Pacific Northwest Evidence-based Practice Center under Contract No. 290-2015-00009-I.). Rockville, MA: Agency for Healthcare Research and Quality; 2019.
43. Rossaro L, Torruellas C, Dhaliwal S, et al. Clinical outcomes of hepatitis C treated with pegylated interferon and ribavirin via telemedicine consultation in Northern California. *Digestive Dis Sci.* 2013;58(12):3620-3625.
44. Harris M, Rhodes T. Injecting practices in sexual partnerships: hepatitis C transmission potentials in a 'risk equivalence' framework. *Drug Alcohol Depend.* Oct 1 2013;132(3):617-623.
45. Batchelder AW, Peyser D, Nahvi S, Arnsten JH, Litwin AH. "Hepatitis C treatment turned me around:" Psychological and behavioral transformation related to hepatitis C treatment. *Drug Alcohol Depend.* 2015;153:66-71.
46. Midgard H, Hajarizadeh B, Cunningham EB, et al. Changes in risk behaviours during and following treatment for hepatitis C virus infection among people who inject drugs: The ACTIVATE study. *Int J Drug Policy.* Sep 2017;47:230-238.

47. Clark JA, Gifford AL. Resolute efforts to cure hepatitis C: Understanding patients' reasons for completing antiviral treatment. *Health.* Sep 2015;19(5):473-489.
48. Madden A, Hopwood M, Neale J, Treloar C. Beyond cure: patient reported outcomes of hepatitis C treatment among people who inject drugs in Australia. *Harm Reduct J.* 2018;15(1):42.
49. Williams BE, Nelons D, Seaman A, et al. Life projects: the transformative potential of direct-acting antiviral treatment for hepatitis C among people who inject drugs. *Int J Drug Policy.* 2019.
50. Kay ES, Batey DS, Mugavero MJ. The HIV treatment cascade and care continuum: updates, goals, and recommendations for the future. *AIDS Res Ther.* 2016;13:35.
51. Rettler H, Klevens M, Haney G, Randall L, DeMaria A, Goderre J. Building health IT capacity to improve HIV infection health outcomes. *Am J Manag Care.* 2016;22(12):821-825.
52. Martin NK, Hickman M, Hutchinson SJ, Goldberg DJ, Vickerman P. Combination interventions to prevent HCV transmission among people who inject drugs: modeling the impact of antiviral treatment, needle and syringe programs, and opiate substitution therapy. *Clin Infectious Dis.* 2013;57 Suppl 2:S39-45.
53. Martin NK, Vickerman P, Grebely J, et al. Hepatitis C virus treatment for prevention among people who inject drugs: Modeling treatment scale-up in the age of direct-acting antivirals. *Hepatol.* 2013;58(5):1598-1609.
54. Fraser H, Martin NK, Brummer-Korvenkontio H, et al. Model projections on the impact of HCV treatment in the prevention of HCV transmission among people who inject drugs in Europe. *J Hepatol.* 2018;68(3):402-411.
55. Bennett H, McEwan P, Sugrue D, Kalsekar A, Yuan Y. Assessing the Long-Term Impact of Treating Hepatitis C Virus (HCV)-Infected People Who Inject Drugs in the UK and the Relationship between Treatment Uptake and Efficacy on Future Infections. *PLoS One.* 2015;10(5):e0125846.
56. Grady BP, Schinkel J, Thomas XV, Dalgard O. Hepatitis C virus reinfection following treatment among people who use drugs. *Clin Infectious Dis.* 2013;57 Suppl 2:S105-110.
57. A B, G vdB, F L. Substantial decline in acute HCV infections among Dutch HIV+ MSM after DAA roll out. Conference on Retroviruses and Opportunistic Infections (CROI); 2017; Seattle.
58. Rolls DA, Sacks-Davis R, Jenkinson R, et al. Hepatitis C transmission and treatment in contact networks of people who inject drugs. *PLoS One.* 2013;8(11):e78286.
59. Hellard M, Rolls DA, Sacks-Davis R, et al. The impact of injecting networks on hepatitis C transmission and treatment in people who inject drugs. *Hepatol.* 2014;60(6):1861-1870.
60. Gelberg L, Andersen RM, Leake BD. The Behavioral Model for Vulnerable Populations: application to medical care use and outcomes for homeless people. *Health Serv Res.* 2000;34(6):1273-1302.
61. Stein JA, Andersen RM, Robertson M, Gelberg L. Impact of hepatitis B and C infection on health services utilization in homeless adults: a test of the Gelberg-Andersen Behavioral Model for Vulnerable Populations. *Health Psychol.* 2012;31(1):20-30.
62. Stein JA, Andersen R, Gelberg L. Applying the Gelberg-Andersen behavioral model for vulnerable populations to health services utilization in homeless women. *J Health Psychol.* 2007;12(5):791-804.
63. Friedmann PD, D'Aunno TA, Jin L, Alexander JA. Medical and psychosocial services in drug abuse treatment: do stronger linkages promote client utilization? *Health Serv Res.* 2000;35(2):443-465.
64. Friedmann PD, Lemon SC, Stein MD. Transportation and retention in outpatient drug abuse treatment programs. *J Subst Abuse Treat.* 2001;21(2):97-103.
65. Friedmann PD, Lemon SC, Stein MD, Etheridge RM, D'Aunno TA. Linkage to medical services in the Drug Abuse Treatment Outcome Study. *Med Care.* 2001;39(3):284-295.
66. Stout RL, Wirtz PW, Carbonari JP, Del Boca FK. Ensuring balanced distribution of prognostic factors in treatment outcome research. *J Stud Alcohol Suppl.* 1994;12:70-75.
67. Bruggmann P, Litwin AH. Models of care for the management of hepatitis C virus among people who inject drugs: one size does not fit all. *Clin Infectious Dis.* 2013;57 Suppl 2:S56-61.

68. Administration SAaMHS. Trauma-Informed Care in Behavioral Health Services. *Treatment Improvement Protocol (TIP) Series 57. HHS Publication No. (SMA) 13-4801.* Rockville, MD: Substance Abuse and Mental Health Services Administration; 2014.
69. Thakarar K, Weinstein ZM, Walley AY. Optimising health and safety of people who inject drugs during transition from acute to outpatient care: narrative review with clinical checklist. *Postgraduate medical journal.* Jun 2016;92(1088):356-363.
70. Begg CB. Suspended judgment. Significance tests of covariate imbalance in clinical trials. *Control Clin Trials.* 1990;11(4):223-225.
71. Senn S. Seven myths of randomisation in clinical trials. *Stat Med.* 2013;32(9):1439-1450.
72. Lewis JA. Statistical principles for clinical trials (ICH E9): an introductory note on an international guideline. *Stat Med.* 1999;18(15):1903-1942.
73. Lewis J, Louv W, Rockhold F, Sato T. The impact of the international guideline entitled Statistical Principles for Clinical Trials (ICH E9). *Stat Med.* 2001;20(17-18):2549-2560.
74. Schulz KF, Altman DG, Moher D, Group C. CONSORT 2010 statement: updated guidelines for reporting parallel group randomized trials. *Ann Intern Medicine.* 2010;152(11):726-732.
75. Kent DM, Rothwell PM, Ioannidis JP, Altman DG, Hayward RA. Assessing and reporting heterogeneity in treatment effects in clinical trials: a proposal. *Trials.* 2010;11:85.
76. Kent DM, Hayward RA. Limitations of applying summary results of clinical trials to individual patients: the need for risk stratification. *JAMA.* 2007;298(10):1209-1212.
77. Chinn S. A simple method for converting an odds ratio to effect size for use in meta-analysis. *Stat Med.* 2000;19(22):3127-3131.
