# Supplementary material for: Mobile Telemedicine for Treating Chronic Hepatitis C Among Rural People Who Inject Drugs: A Randomized Clinical Trial
Source: JAMA Netw Open. 2026 Jan 26;9(1):e2555125. doi: 10.1001/jamanetworkopen.2025.55125 (PMC12836132; doi:10.1001/jamanetworkopen.2025.55125)
Supplement: Supplement 2. — eTable 1. Schedule of Participant Payments eTable 2. Baseline Characteristics Comparing Randomized Participants With and Without Follow-Up Data, Rural Northern New England, 2022 to 2024 eTable 3. Viral Clearance at 12-Week Follow-Up by Study Condition, Initiation, and Completion of Direct-Acting Antiviral (DAA) Treatment, Rural Northern New England, 2022 to 2024 [file jamanetwopen-e2555125-s002.pdf]

Supplementary Online Content

Friedmann PD, Wilson D, de Gijsel D, et al. Mobile telemedicine for treating chronic hepatitis C among rural people who inject drugs: a randomized clinical trial. *JAMA Netw Open*. 2026;9(1):e2555125. doi:10.1001/jamanetworkopen.2025.55125

eTable 1. Schedule of Participant Payments

eTable 2. Baseline Characteristics Comparing Randomized Participants With and Without Follow-Up Data, Rural Northern New England, 2022 to 2024

eTable 3. Viral Clearance at 12-Week Follow-Up by Study Condition, Initiation, and Completion of Direct-Acting Antiviral (DAA) Treatment, Rural Northern New England, 2022 to 2024

This supplementary material has been provided by the authors to give readers additional information about their work.

© 2026 Friedmann PD et al. *JAMA Network Open*.

eTable 1. Schedule of participant payments.	
Tested negative for HCV antibody at Visit T0, or otherwise excluded.	$10
Tested positive for HCV antibody, had a venous blood draw performed and completed enrollment at Visit T0.	$20
Attended Visit T1, ineligible for randomization. Excluded, received laboratory results and appropriate referrals.	$10
Attended Visit T1, eligible for randomization and treatment, completed the baseline ACASI interview.	$20
Attended brief week 4 check-in visit	$20
Attended "end-of-treatment visit" (week 8 or 12 after treatment initiation Visit T1, or week 16 if treatment not initiated)	$40
Attended 12 week follow-up (week 12 after "end-of-treatment" or 28 weeks after Visit T1 if treatment not initiated)	$40
Attended 24 week follow-up (week 24 after "end-of-treatment" or j40 weeks after Visit T1 if treatment not initiated)	$40
Attended 36 week follow-up (week 36 after "end-of-treatment" or 52 weeks after Visit T1 if treatment not initiated)	$60
Referred a potentially eligible participant who completed the enrollment (T0) visit, whether or not they were eventually eligible for randomization and treatment.	$5

© 2026 Friedmann PD et al. *JAMA Network Open*.

eTable 2. Baseline characteristics comparing randomized participants with and without follow-up data, rural northern New England, 2022 to 2024

	Has any follow-up data?	
	**No**	**Yes**
All randomized participants	31 (20.8)	119 (79.3)
Site, N (%)		
Cheshire County (Keene), NH	14 (27.5)	37 (72.6)
Windham County (Brattleboro), VT	10 (12.1)	73 (88.0)
Bennington County (Bennington), VT	7 (43.85)	9 (56.3)
Age, mean (std deviation)	35.3 (6.42)	38.8 (8.40)
Gender, N (%)		
Male	24 (23.3)	79 (76.7)
Female	5 (11.4)	39 (88.6)
Other		1 (100)
Missing	2 (100)	
Race, N (%)		
Asian, Pacific Islander		1 (100)
Black		2 (100)
Mixed race	2 (25.0)	6 (75.0)
White	27 (20.2)	107 (79.9)
Other*		3 (100
Missing	2 (100)	
Hispanic, N (%)		
Yes	2 (25.0)	6 (75.0)
No	27 (19.6)	111 (80.4)
Don't know		2 (100
Missing	2 (100)	
Education, N (%)		
Less than high school	9 (20.9)	34 (79.1)
High school diploma or GED	12 (17.1)	58 (82.9)
Some college	6 (23.1)	20 (76.9)
Associates degree, trade or technical	1 (20.0)	4 (80.0)
College degree or more	1 (33.3)	2 (66.7)
Missing	2 (100)	1 (100)
Health insurance coverage, N (%)		
Yes	27 (19.2)	114 (80.9)
No	1 (25.0)	3 (75.0)
Don't know	1 (33.3)	2 (66.7)
Missing	2 (100)	
Unhoused in past 6 months, N (%)		
Yes	22 (21.0)	83 (79.0)
No	6 (14.3)	36 (85.7)
Missing/Refused	3 (100)	

© 2026 Friedmann PD et al. *JAMA Network Open*.

Ever overdosed, N (%)		
Yes	22 (21.6)	80 (78.4)
No	7 (15.2)	39 (84.8)
Missing	2 (100)	
Injected within 30 days of baseline, N (%)		
Yes	16 (16.5)	81 (83.5)
No	13 (25.5)	38 (74.5)
Missing	2 (100)	
MOUD within 30 days of baseline, N (%)		
Yes	13 (23.6)	42 (76.4)
No	15 (16.9)	74 (83.2)
Don't know		3 (100)
Missing	3 (100)	
Reported shared syringes/equipment within 30 days of baseline, N (%)		
Yes	7 (24.1)	22 (75.9)
No	21 (17.8)	97 (82.2)
Missing	3 (100)	
Any opioid use within 30 days of baseline, N (%)		
Yes	18 (17.1)	87 (82.9)
No	11 (25.6)	32 (74.4)
Missing	2 (100)	

Abbreviations. EUC: Enhanced Usual Care; MTC: Mobile Telemedicine HCV Care; MOUD: Medication for Opioid Use Disorder

*In the Other category, two individuals reported “Hebrew” and one “Italian.”

© 2026 Friedmann PD et al. *JAMA Network Open*.

eTable 3. Viral clearance at 12-week follow-up* by study condition, initiation, and completion of direct-acting antiviral (DAA) treatment, rural northern New England, 2022 to 2024				
		Viral Clearance*		
	Row Total	Yes	No	Missing/Not Obtained
Mobile HCV Telemedicine Care [MTC], n (%)	75	28 (37.3)	25 (33.3)	24 (32.0)
Did not initiate DAA treatment	13	1 (7.7)	10 (76.9)	2 (15.4)
Initiated DAA treatment	43	27 (62.8)	12 (27.9)	4 (9.3)
Initiated but did not complete DAA treatment	9	4 (44.4)	4 (44.4)	1 (11.1)
Completed DAA treatment	34	23 (67.7)	8 (25.5)	3 (8.8)
Missing initiation or completion status	19	0	1 (5.3)	18 (94.7)
Enhanced Usual Care [EUC], n (%)	75	14 (18.7)	45 (60.0)	16 (21.3)
Did not initiate DAA treatment	42	1 (2.4)	38 (90.5)	3 (7.1)
Initiated DAA treatment	20	13 (65.0)	6 (30.0)	1 (5.0)
Initiated but did not complete DAA treatment	5	2 (40.0)	3 (60.0)	0
Completed DAA treatment	15	11 (73.3)	3 (20.0)	1 (6.7)
Missing initiation or completion status	13	0	1	12

*Viral clearance was assessed as negative HCV RNA at the 12-week follow-up after treatment completion (i.e., sustained viral response at 12 weeks [SVR12] among treated participants) or week 28 after randomization (Visit T1) among participants who did not initiate treatment.

© 2026 Friedmann PD et al. *JAMA Network Open*.
