# Supplementary material for: Mobile Telemedicine for Treating Chronic Hepatitis C Among Rural People Who Inject Drugs: A Randomized Clinical Trial
Source: JAMA Netw Open. 2026 Jan 26;9(1):e2555125. doi: 10.1001/jamanetworkopen.2025.55125 (PMC12836132; doi:10.1001/jamanetworkopen.2025.55125)
Supplement: Supplement 3. — Data Sharing Statement [file jamanetwopen-e2555125-s003.pdf]

Data Sharing Statement

Friedmann. Mobile Telemedicine for Treating Chronic Hepatitis C Among Rural People Who Inject Drugs. *JAMA Netw Open*. Published January 26, 2026.
doi:10.1001/jamanetworkopen.2025.55125

Data
Additional Information: ClinicalTrials.gov Identifier: NCT05466331
Data available: Yes
Data types: Deidentified participant data, Data dictionary
How to access data: The data that support the findings of this study are available on request from ruralopioids@uw.edu.
When available: With publication

Supporting Documents
Document types: None

Additional Information
Who can access the data: Anyone requesting the data
Types of analyses: For any purpose
Mechanisms of data availability: Without investigator support
